# Supplementary material for: Pretreatment-free SERS sensing of microplastics using a self-attention-based neural network on hierarchically porous Ag foams
Source: Nat Commun. 2024 May 28;15:4351. doi: 10.1038/s41467-024-48148-w (PMC11133413; doi:10.1038/s41467-024-48148-w)
Supplement: Supplementary file 1 — Supplementary Information [file 41467_2024_48148_MOESM1_ESM.docx]

**Supplementary Information**

**Pretreatment-free SERS sensing of microplastics using a self-attention-based neural network on hierarchically porous Ag foams**

Olga Guselnikova^1,2*^, Andrii Trelin^3^, Yunqing Kang^1,4^, Pavel Postnikov^2,3^, Makoto Kobashi^4^,

Asuka Suzuki^4^, Lok Kumar Shrestha^1,5^, Joel Henzie^1*^, Yusuke Yamauchi^4,6*^

^1^ National Institute for Materials Science (NIMS), Tsukuba, Ibaraki, Japan

^2^ Research School of Chemistry and Applied Biomedical Sciences, Tomsk Polytechnic University, Tomsk, Russian Federation

^3^ Department of Solid-State Engineering, University of Chemistry and Technology, Prague, Czech Republic

^4^ Department of Materials Process Engineering, Graduate School of Engineering, Nagoya University, Furo-cho, Chikusa-ku, Nagoya, Japan

^5^ Department of Materials Science, Institute of Pure and Applied Sciences, University of Tsukuba, Tennoda, Tsukuba, Japan

^6^ Australian Institute for Bioengineering and Nanotechnology (AIBN), The University of Queensland, Brisbane, QLD, Australia

**Table of Content**

**Supplementary figures**

**Suppl. Fig. 1.** Linear sweep voltammetry curve of a solution containing AgNO_3_ (40 mM, 0.5 mL), PS_18,000_-*b*-PEO_7500_ THF (5 mg mL^-1^, 2 mL), EtOH (1mL) and H_2_O (3 mL).

**Suppl. Fig. 2.** **a** Reconstructed three-dimensional (3D) µ-CT scan of AgF@AgM@C10, **b** macropore size distribution showing average pore size of 262 µm, **c** porosity distribution versus thickness with an average porosity of 84.1%, and **d** flow velocity analysis over the AgF thickness.

**Suppl. Fig. 3.** Oxide stripping curves of **a** AgF@AgM and **b** AgF.

**Suppl. Fig. 4.** XPS spectra of AgF@AgM and AgF@AgM@C10. **a**, **c** Survey spectra with an inset describing sample composition in atomic %. **b**, **d** High-resolution spectra of the Ag 3*d* peaks.

**Suppl. Fig. 5.** Raman spectra of AgF@AgM, AgF@AgM@C10 and ADT-C_10_.

**Suppl. Fig. 6.** Wettability study of AgF, AgF@AgM, and AgF@AgM@C10.

**Suppl. Fig. 7.** Cross-section SEM of AgF@AgM@C10 after circulating 10^5^ MPS L^-1^ PS suspension for 30 min.

**Suppl. Fig. 8.** Adsorption capacity of AgF, AgF@AgM, AgF@AgM@C10, AgF@AgM@COOH, and Klarite (*- only the weight of gold layer on Si is considered for capacity calculations) for PS (30 µm) measured gravimetrically after circulating 10^5^ MPs L ^-1^ suspension over each sample for 30 min.

**Suppl. Fig. 9.** SERS spectra of AgF@AgM@COOH.

**Suppl. Fig. 10.** Results of the digital water flow simulation from VGStudio Max on **a** AgF and **b** AgF@AgM. **c**, **d** Permeability and average fluid velocity of AgF and AgF@AgM.

**Suppl. Fig. 11.** Experimental UV-Vis spectra of AgF and AgF@AgM.

**Suppl. Fig. 12.** Simulated electromagnetic field intensity distributions using plane wave excitation with the electric field rotated to 45° polarization.

**Suppl. Fig. 13.** 3D SERS map of AgF@AgM@C10 after deposition of MB (584 × 584 × 60 μm^3^).

**Suppl. Fig. 14.** Stability tests for AgF@AgM@C10 and AgF@AgM towards oxidation in air.

**Suppl. Fig. 15.** Stability tests for AgF@AgM@C10 and AgF@AgM towards oxidation in water.

**Suppl. Fig. 16.** Explanation of the choice of microplastics (MPs).

**Suppl. Fig. 17.** SEM images and size distribution histograms of MPs used in this study (**a** PS – beads and fragments, **b** PE, **c** PMMA, **d** PTFE, **e** Nylon, **f** PET (diameter of the fiber is 20±2 µm).

**Suppl. Fig. 18.** Characterization of AgM@C10 for further comparison with AgF@AgM@C10.

**Suppl. Fig. 19.** Comparison of EF for PE, PMMA, PTFE, Nylon, and PET obtained on AgF@AgM@C10 at 532 nm.

**Suppl. Fig. 20.** Comparison of EF for PS, PE, PMMA, Nylon, PTFE, and PET obtained on AgM@C10 at 532 nm.

**Suppl. Fig. 21.** Comparison of EF for PS, PE, PMMA, Nylon, PTFE, and PET obtained on Klarite at 785 nm and comparison of EFs for MPs on AgF@AgM@C10, AgM@C10, and Klarite.

**Suppl. Fig. 22. a** Photograph of the AgF@AgM@C10 structure in the μ-Slide I Luer cell.

**Suppl. Fig. 23.** **a** SERS spectra and **b** 2D maps of AgF@AgM@C10 substrates exposed to PS and PE collected in a 0.1×0.1 mm^2^ area (0.5 µm step).

**Suppl. Fig. 24.** Comparison of the performance of various convolutional architectures (ResNet, AlexNet, DenseNet) in terms of F1 scores (*i.e.*, a balanced parameter between precision and recall) for MPs detection.

**Suppl. Fig. 25.** An illustration summarizing the types of sample types analyzed with SERS and subsequently subjected to the neural network for identification.

**Suppl. Fig. 26.** A confusion matrix describing the performance of SpecATNet with single-component MP samples (0.15 mg L^-1^).

**Suppl. Fig. 27.** Accuracies of SpecATNet predictions for **a** single MP sample, **b** multicomponent samples, and **c** a comparison of the accuracy obtained from 2, 3, 4, and 5 component MPs mixtures by SpecATNet and analogous NN without the self-attention layer, with *p*-values of 0.99, 0.69, 6.9×10-3, and 1.66×10^-6^, respectively (independent samples one-sided T-test, *n* = 29).

**Suppl. Fig. 28.** The photodegradation of different MPs *via* the Fenton reaction.

**Suppl. Fig. 29**. Comparison of EFs for degraded PS_deg_, PE_deg_, PMMA_deg_, Nylon_deg_, PTFE_deg_, and PET_deg_ on the glass.

**Suppl. Fig. 30.** **a** SEM images of dried algae and **b** microscopical images of grown algae in Guillard’s (F/2) Marine Water.

**Suppl. Fig. 31.** Examining the antifouling properties of different substrates.

**Suppl. Fig. 32.** Raman spectra of algae, humic acid, sediments, soil, BSA, and NaCl in comparison with 6 types of MP.

**Suppl. Fig. 32.** Raman spectra of algae, humic acid, sediments, soil, BSA, and NaCl in comparison with 6 types of MP.

**Suppl. Fig. 34. a** Accuracies of SpecATNet prediction for multi-component samples in complex matrices (PS_fr_ corresponds to PS fragments) and **b** matrices without MPs.

**Suppl. Fig. 35.** **a** Dependence of accuracy on the number of spectra in input spectral set for PS/PET and PMMA/PS_fr_ in complex matrices (PS_fr_ corresponds to PS fragments) and **b** comparison of the F1 score of SpecATNet versus a NN without using the self-attention.

**Suppl. Fig. 36.** Timeline of **a** Pyr-GCMS and **b** SERS-SpecATNet-assisted analysis of environmental MPs samples.

**Suppl. Fig. 37.** **a** Overview of the precision, recall and F1 score based on all the samples analyzed in this study on AgF@AgM@C10 and **b** schematic explanation of true positive true negative false positive, and false negative.

**Suppl. Fig. 38**. **a** Precision-recall curve and **b** receiver operating characteristic curve for six types of MPs.

**Suppl. Fig. 39.** Comparison of F1 scores obtained from logical regression model (Log Reg), decision tree model (Dec Tree), support vector machine (SVM), and SpecATNet.

**Suppl. Fig. 40.** Comparison of freshly prepared and after 1 month air storage AgF@AgM@C10 performance for sensing MPs mixtures.

**Suppl. Fig. 41.** Possible extension to other mesoporous Au and Cu depositions on Ni foam (NiF@AuM and NiF@CuM) to reduce the price and preliminary data on SERS sensing of MPs on NiF@AuM and NiF@CuM at 785 nm.

**Supplementary Tables**

**Suppl. Table 1.** Comparative table of visual, mass, and optical spectroscopic strategies for MPs analysis.

**Suppl. Table 2.** Assigning bond vibrations to the Raman and SERS spectra of ADT-C_10_ and AgF@AgM@C10.

**Suppl. Table 3**. Assigning bond vibrations to the Raman spectra of PS, PE, PMMA, Nylon, and PTFE.

**Suppl. Table 4.** Conversion of MP concentrations used in this study.

**Suppl. Table 5.** Comparison of different approaches for identification of the chemical structure of MPs.

**Suppl. Table 6.** Information about the collected spectra and their amount used in this study.

**Suppl. Table 7.** Composition of groundwater-certified reference material ERM-CA616.

**Suppl. Table 8.** Calculation of cost of porous plasmonic substrate for 1 cm^2^ of foam (starting material only, based on prices on 30.10.2023).

**Supplementary References**


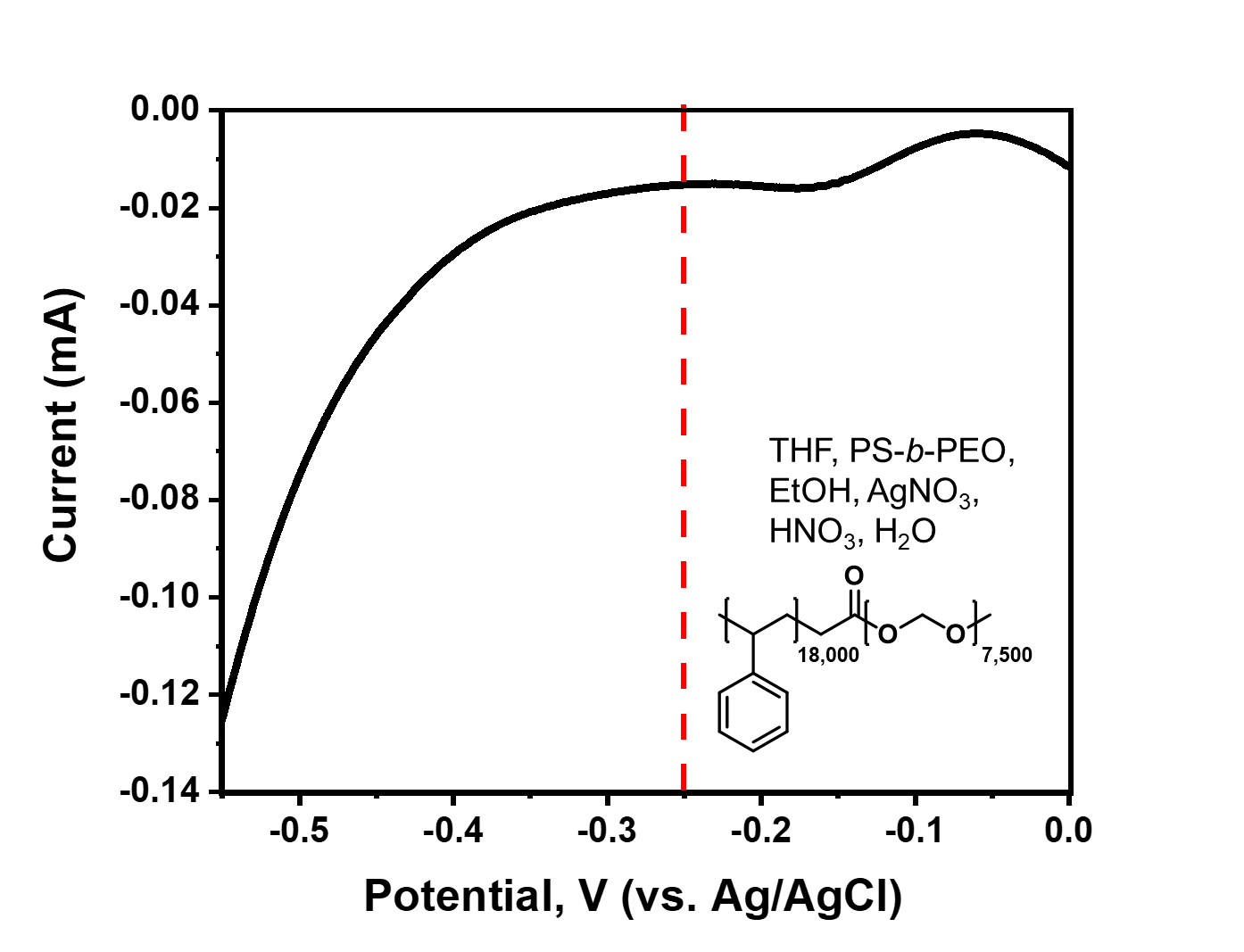


**Suppl. Fig. 1.** Linear sweep voltammetry curve of a solution containing AgNO_3_ (40 mM, 0.5 mL), PS_18,000_-*b*-PEO_7500_ THF (5 mg mL^-1^, 2 mL), EtOH (1mL) and H_2_O (3 mL). According to previous studies,^1^ the potential to form a mesoporous structure should be chosen in the Ag reduction region before the hydrogen evolution reduction potential. In this study, the potential -0.25 V is chosen, where the mesoporous ordered structure is obtained.


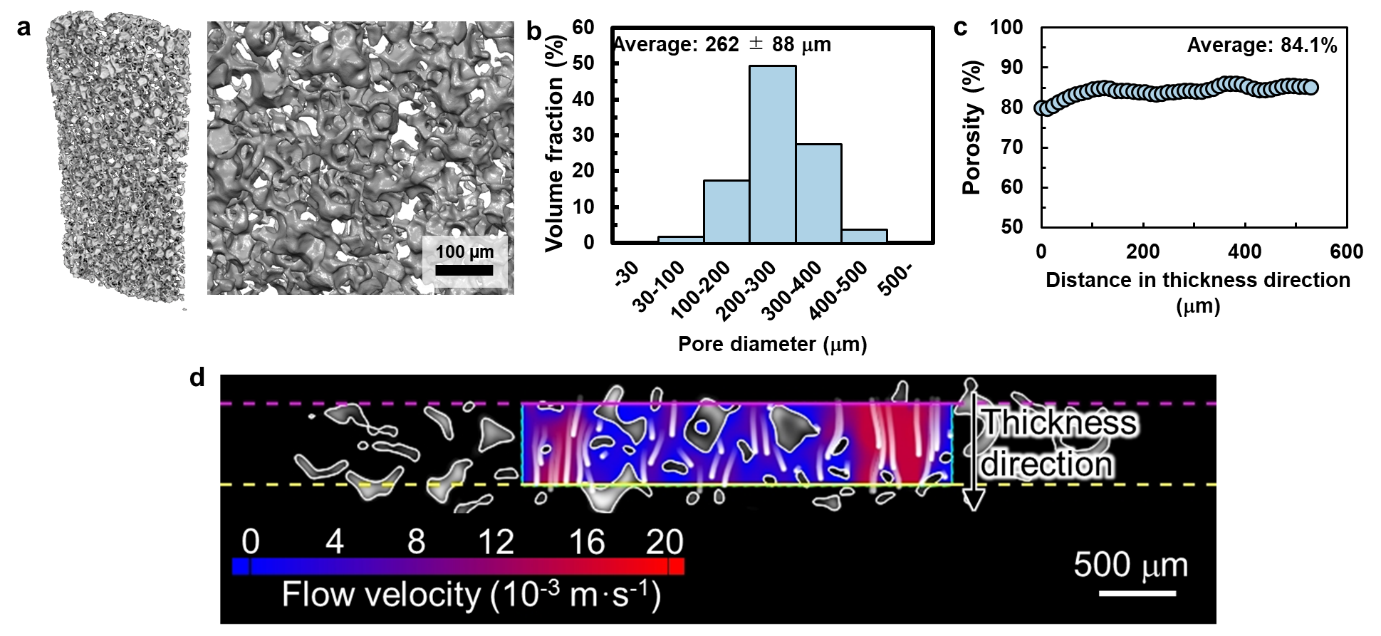


**Suppl. Fig. 2.** **a** Reconstructed three-dimensional (3D) µ-CT scan of AgF@AgM@C10, **b** macropore size distribution showing average pore size of 262 µm, **c** porosity distribution versus thickness with an average porosity of 84.1%, and **d** flow velocity analysis over the AgF thickness. The color in **c** shows the flow velocity inside AgF@AgM@C10. The number of pores analyzed in **b** is 36, and the 95% confidence interval is ±88 µm from the average pore size.


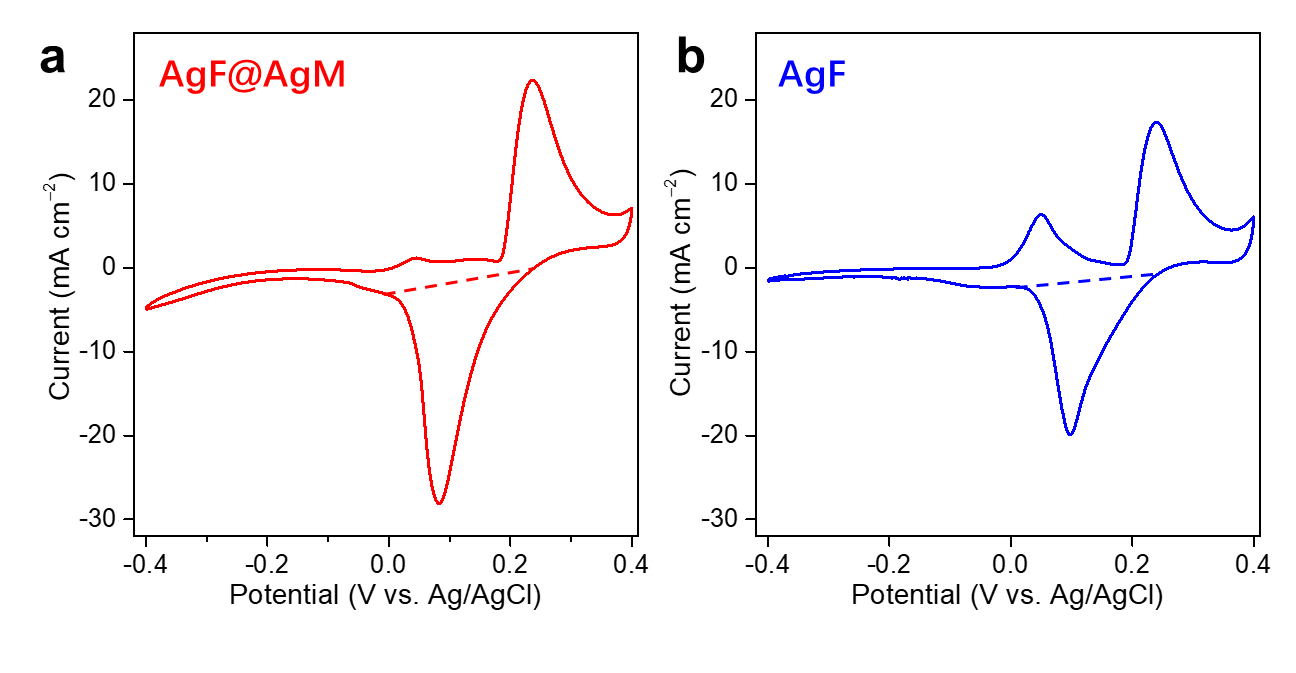


**Suppl. Fig. 3.** Oxide stripping curves of **a** AgF@AgM and **b** AgF. The dashed line indicates the region selected for calculating the redox peak size, which is further used to estimate ECSA.


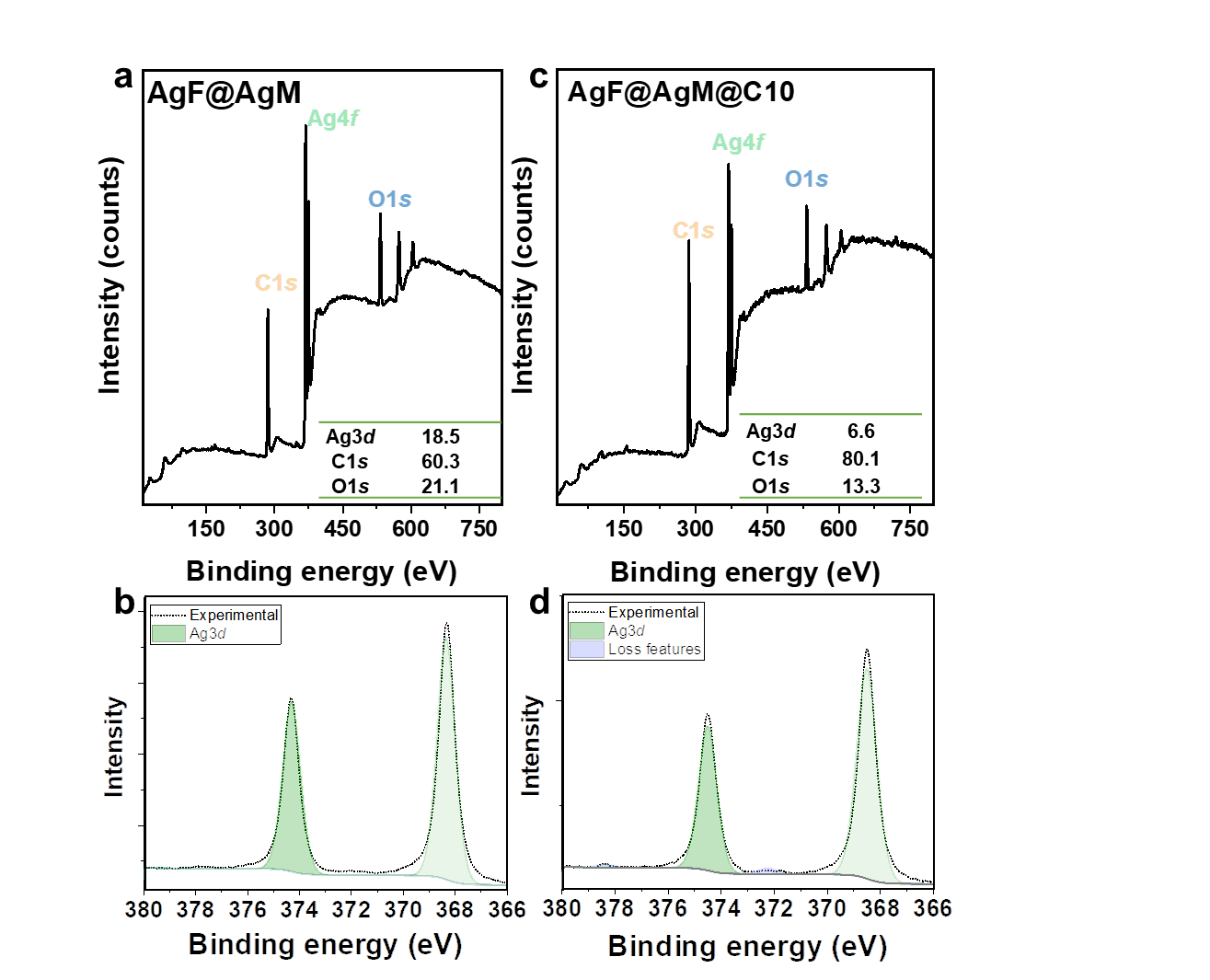


**Suppl. Fig. 4.** XPS spectra of AgF@AgM and AgF@AgM@C10. **a**, **c** Survey spectra with an inset describing sample composition in atomic %. **b**, **d** High-resolution spectra of the Ag 3*d* peaks. For AgF@AgM, the peak positions of Ag 3*d*_5/2_ and 3*d*_3/2_ (368.3 and 374.3 eV) correspond to Ag(0) without the oxidized states.^2^ Samples coated with 4-decylphenyl groups (AgF@AgM@C10) remain metallic and show no oxidation features according to the Ag 3*d* region in XPS.^2,3^ There is a slight attenuation of the Ag 3*d* peak, indicating the coating of Ag by the organic layer.

***Supplementary Note 1 to Suppl. Fig. 4 on calculation of 4-decylphenyl layer thickness by XPS.***

The thickness of the organic layer was estimated from the surface chemical composition and signal intensities reported in Suppl. Table 1 and Fig. 2b.^4^ The Ag signal (Ag 3*d*_5/2_ is chosen, 68.3 eV) is observed after grafting of 4-decylphenyl groups and covering the initial AgF@AgM surface. This attenuation of the Ag-related signal can be used to calculate the organic layer thickness using the following equation (1):

*I*/*I*_0_ = exp(-*d*/*λ* sin *θ*) eq. (1)

where *d* is the layer thickness, *λ* is the mean free path of photoelectrons in the organic layer, *θ* is the analysis takeoff angle relative to the surface (90° from the plain surface), and *I*/*I*_0_ is the ratio of the Ag 3*d*_5/2_ peak intensities. The value of *λ* (mg m^-2^) is deduced from the empirical formula derived by Seah and Dench,^5^ eq. (2):

*λ*_k_ = *A*_n_/*E*_k_^2^ + *B*_n_*E*_k_^1/2^, eq. (2)

where *E*_k_ (eV) is the kinetic energy of photoelectrons. For an Al Kα source *E*_k_ = 1486.6 – *E*_B_ = 1486.6 – 68.3 = 1418.3 eV. If the substrate is coated with organic materials, *A*_n_ = 49 and *B*_n_ = 0.11.

*λ*_k_ = *A*_n_/*E*_k_^2^ + *B*_n_*E*_k_^1/2^ = 49/(1418.3)^2^ + 0.11×(1418.3)^1/2^ = 4.14 mg m^-2^.

To convert *λ* into nm units, *λ* (mg m^-2^) is divided by the density of the overlayers, estimated here to be 1 g cm^-3^. For Ag 3*d*_5/2_, *λ*_k_ is calculated as 4.14 mg m^-2^ and the average 4-dedcylphenyl thickness is found to be 2.44 nm.


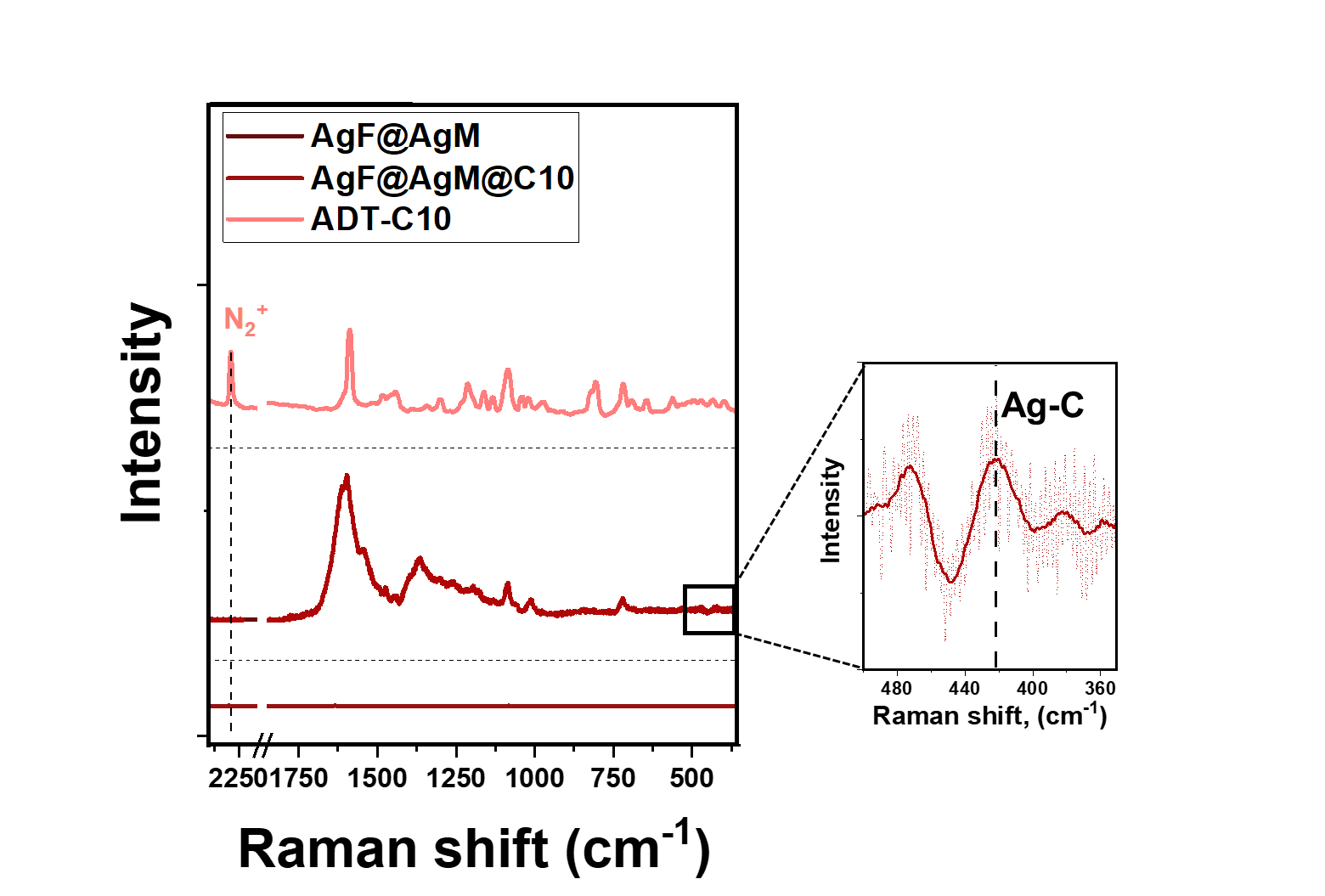


**Suppl. Fig. 5.** Raman spectra of AgF@AgM, AgF@AgM@C10 and ADT-C_10_. AgF@AgM@C10 is prepared *via* covalent modification with 4-decylbenzenediazonium tosylate (ADT-C_10_) in water at 23 °C for 1 h. The ADT-C10 molecule has an N≡N stretching vibration at 2290 cm^-1^, which disappears after interaction with the Ag surface, indicating the attachment of 4-decylphenyl groups. New peaks after functionalization are described in more detail in Suppl. Table 1. The low-intensity bands at ≈420 cm^−1^ correspond to the [covalent](https://www.sciencedirect.com/topics/chemistry/covalent-bond) Ag−C bond, which indicates that the 4-decylphenyl groups are covalently bonded to the Ag surface.^6^


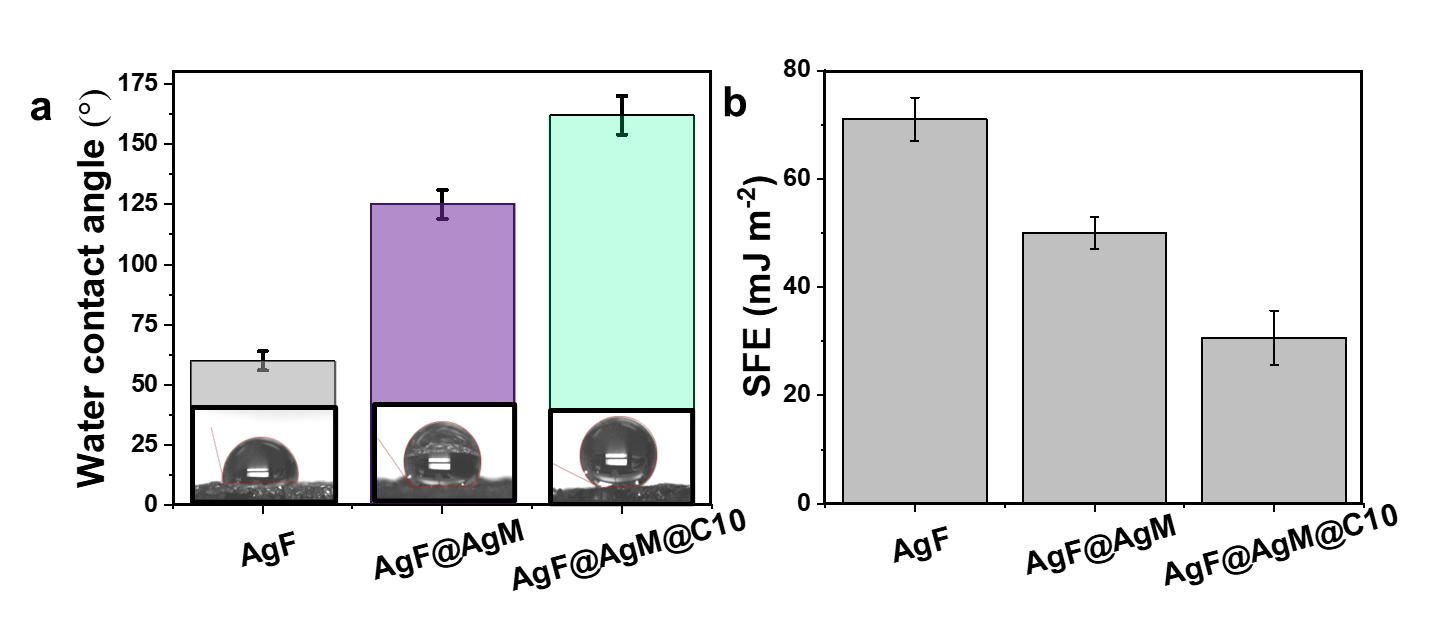


**Suppl. Fig. 6.** Wettability study of AgF, AgF@AgM, and AgF@AgM@C10. **a** Water contact angle (WCA) with an insert of photos taken from goniometer, and **b** surface free energy calculated from OWRK model^7^ using contact angles measured with water and ethylene glycol. The mean value, calculated from three measurements on three samples (*N* = 3, *n* = 3), is presented.

***Supplementary Note 2 to Suppl. Fig. 6.***

The surface wetting properties were tested to estimate the possible interaction of the SERS surface with the targeted hydrophobic MPs. First, the wettability (contact angle – CA) tests were performed using water (Suppl. Fig. 6a) and ethylene glycol, and then surface free energy (SFE) was calculated according to Owens, Wendt, Rabel and Kaelble (OWRK) model.^7^ While AgF shows a CA of 60° and a SFE of 70 mJ m^-2^, the deposition of a mesoporous Ag layer transforms the surface to hydrophobic with a 125° contact angle, along with a decreased SFE. The hydrophobic surface is attributed to its special micro-nanostructure roughness leading to low surface energy property. Functionalized AgF@AgM@C10 becomes superhydrophobic with WCA of 162° and SFE of 30.6 mJ m^-2^, because the introduction of long aliphatic chains commonly leads to the decrease of SFE.^8^ Meanwhile, low energy substance contributes to a lower tendency for water to adhere to it, which is beneficial for prolonging protecting time. As the surface free energy of common MPs is quite low and ranges from 30.1 to 40 mJ m^-2^ ^9^, the superhydrophobic surface readily interacts with MPs via hydrophobic forces.


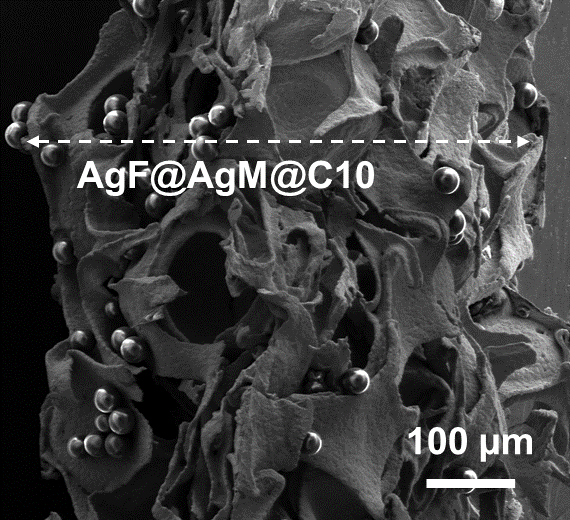


**Suppl. Fig. 7.** Cross-section SEM of AgF@AgM@C10 after circulating 10^5^ MPS L^-1^ PS suspension for 30 min. PS beads penetrate throughout the entire foam and are randomly distributed within it.


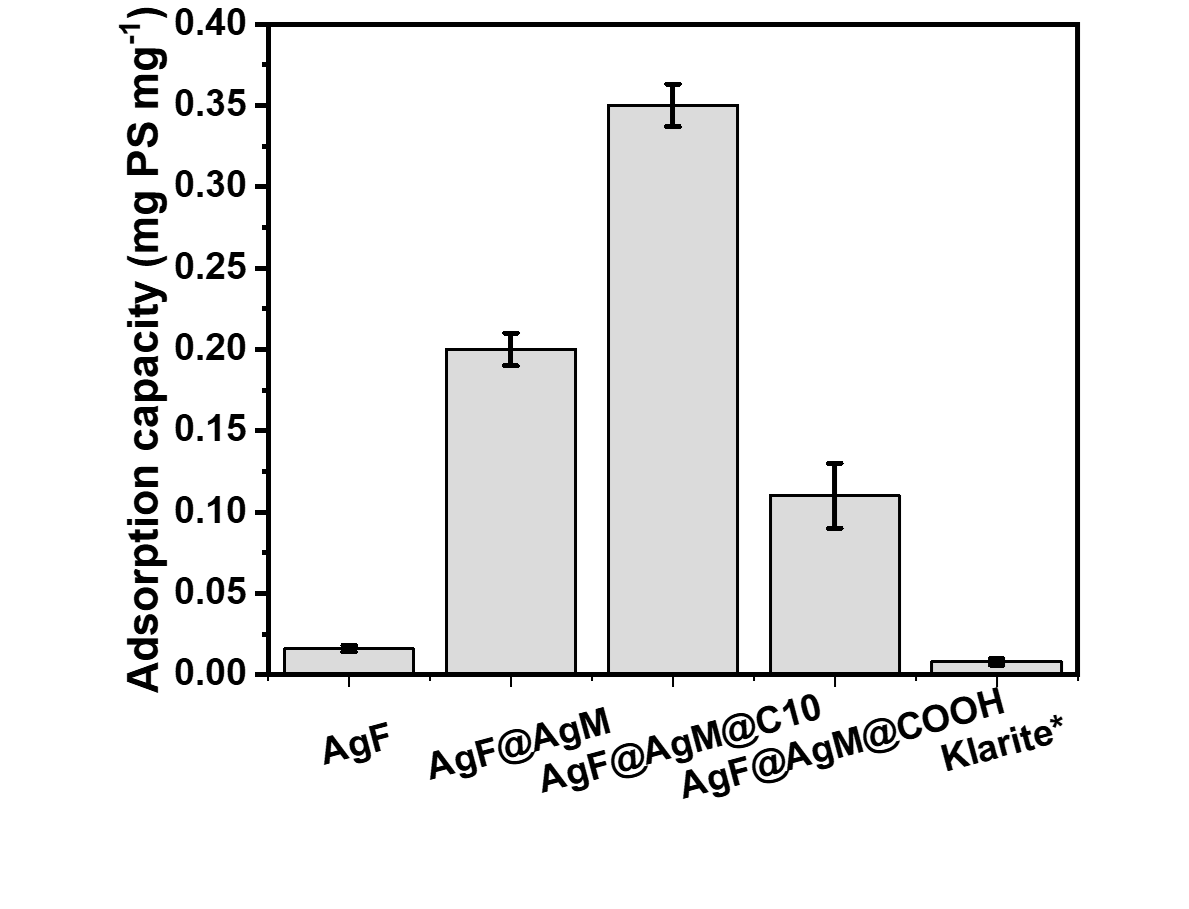


**Suppl. Fig. 8.** Adsorption capacity of AgF, AgF@AgM, AgF@AgM@C10, AgF@AgM@COOH, and Klarite (*- only the weight of gold layer on Si is considered for capacity calculations) for PS (30 µm) measured gravimetrically after circulating 10^5^ MPs L ^-1^ suspension over each sample for 30 min. The AgF substrate has low adsorption capacity due to weak capillary forces. AgF@AgM substrates with rough mesoporous surface structures absorb ≈10 times more PS MPs. Coating the AgF@AgM with a 4-decylphenyl layer to make the AgF@AgM@C10 substrate increases adsorption capacity by an additional ≈2×. On the other hand, adding hydrophilic groups to make the AgF@AgM@COOH substrate decreases the adsorption capacity of the macroporous-mesoporous foams, demonstrating the importance of hydrophobic forces and capillary forces. The mean value, calculated from three measurements on three samples (*N* = 3, *n* = 3), is presented.


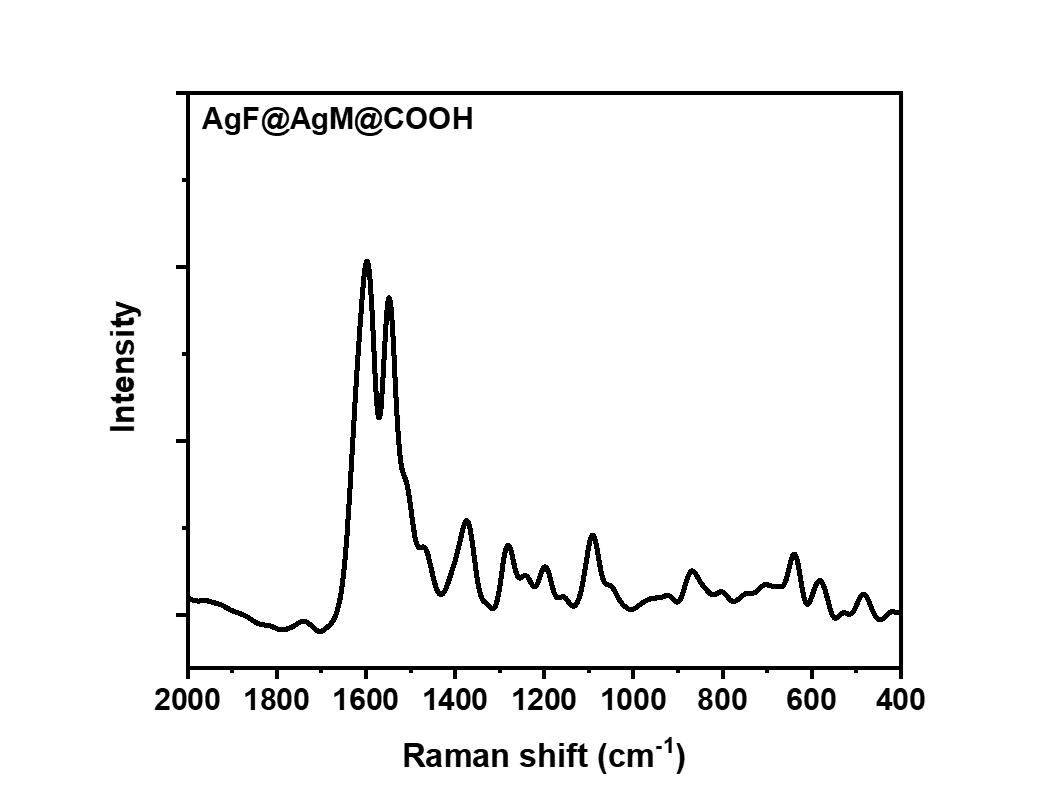


**Suppl. Fig. 9.** SERS spectra of AgF@AgM@COOH. The successful attachment of carboxylic groups is confirmed due to the presence of peaks at 1593 cm^-1^ (C=O stretch), 1542 cm^-1^ (C-C stretch of aromatic ring), 1370 cm^-1^ (OH in-plane bending), 1278 cm^-1^ (C-O stretch, =C-H in-plane deformations), 1087 cm^-1^ (C-O stretch, =C-H in-plane deformation), 862 cm^-1^ (=C-H out-of-plane deformation), and 634 cm^-1^ (=C-H out-of-plane deformation).


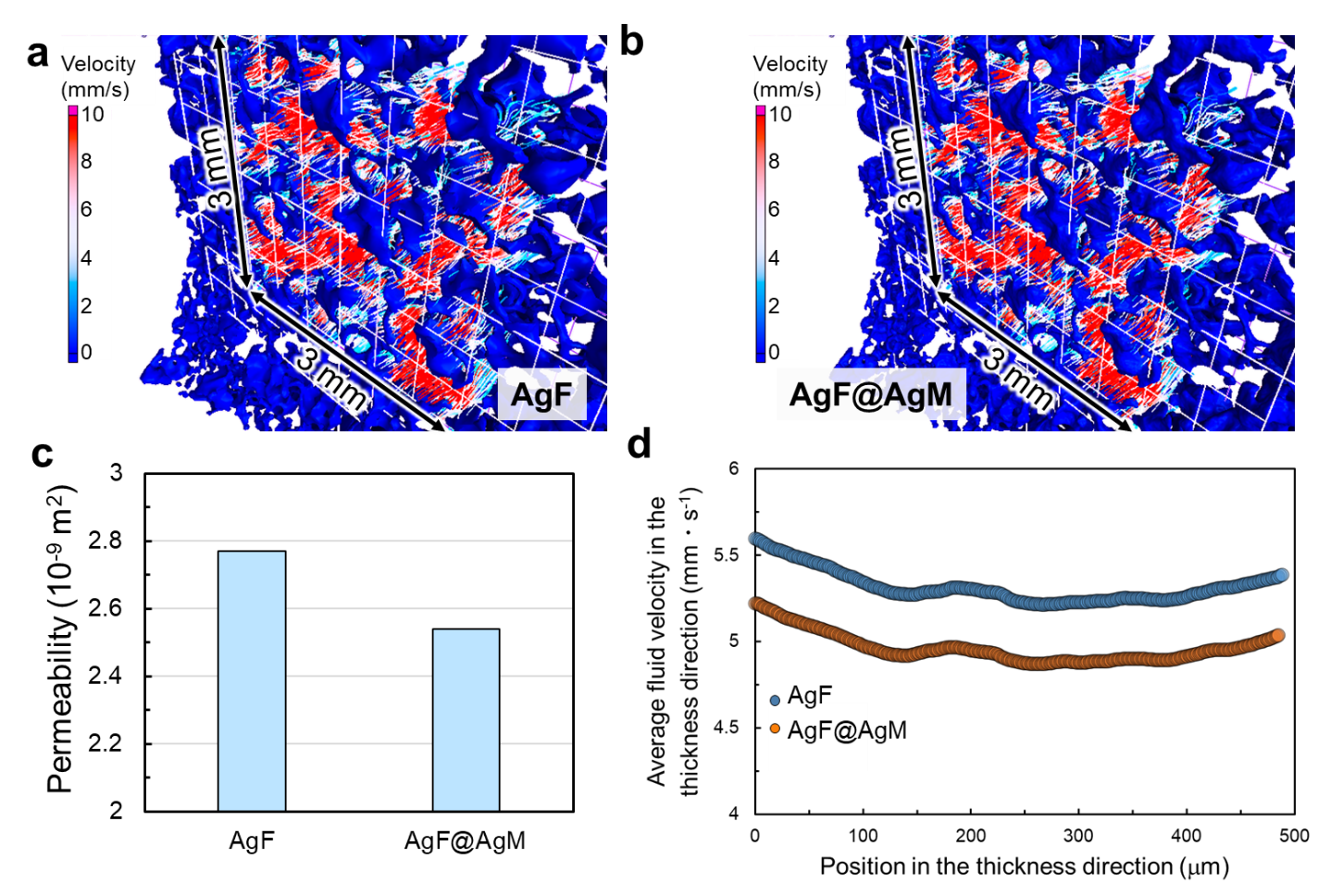


**Suppl. Fig. 10.** Results of the digital water flow simulation from VGStudio Max on **a** AgF and **b** AgF@AgM. **c**, **d** Permeability and average fluid velocity of AgF and AgF@AgM. The thickness direction represents the direction from the back to the front of the 3D images (**a** and **b**).


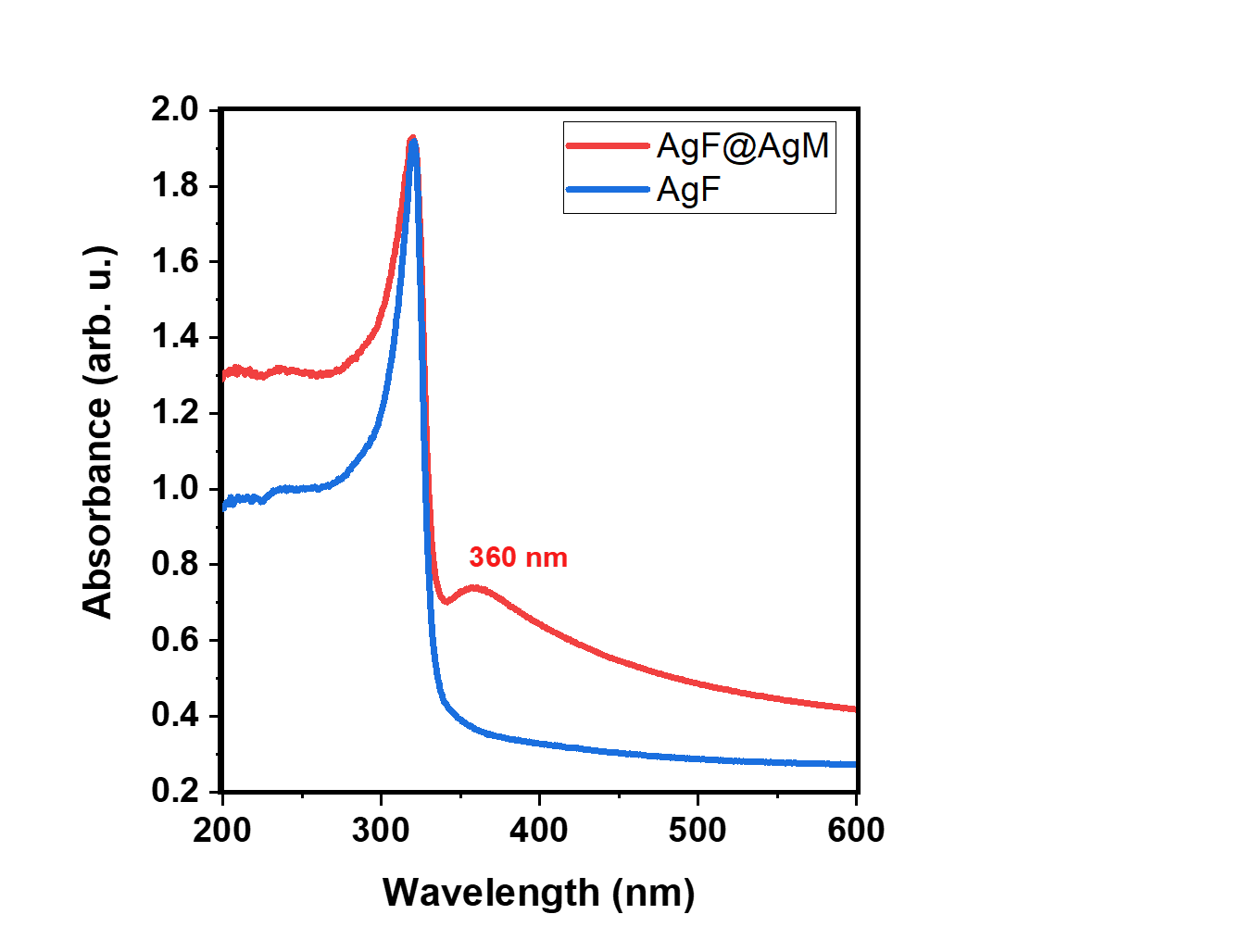


**Suppl. Fig. 11.** Experimental UV-Vis spectra of AgF and AgF@AgM.


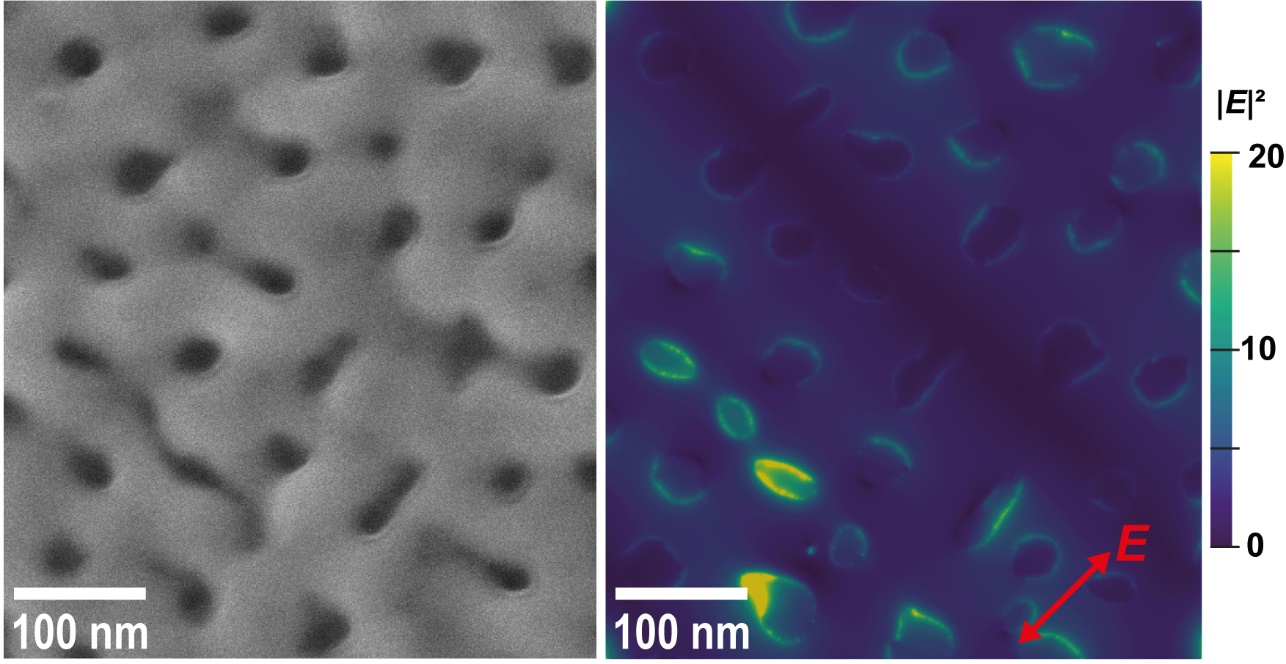


**Suppl. Fig. 12.** Simulated electromagnetic field intensity distributions using plane wave excitation with the electric field rotated to 45° polarization.


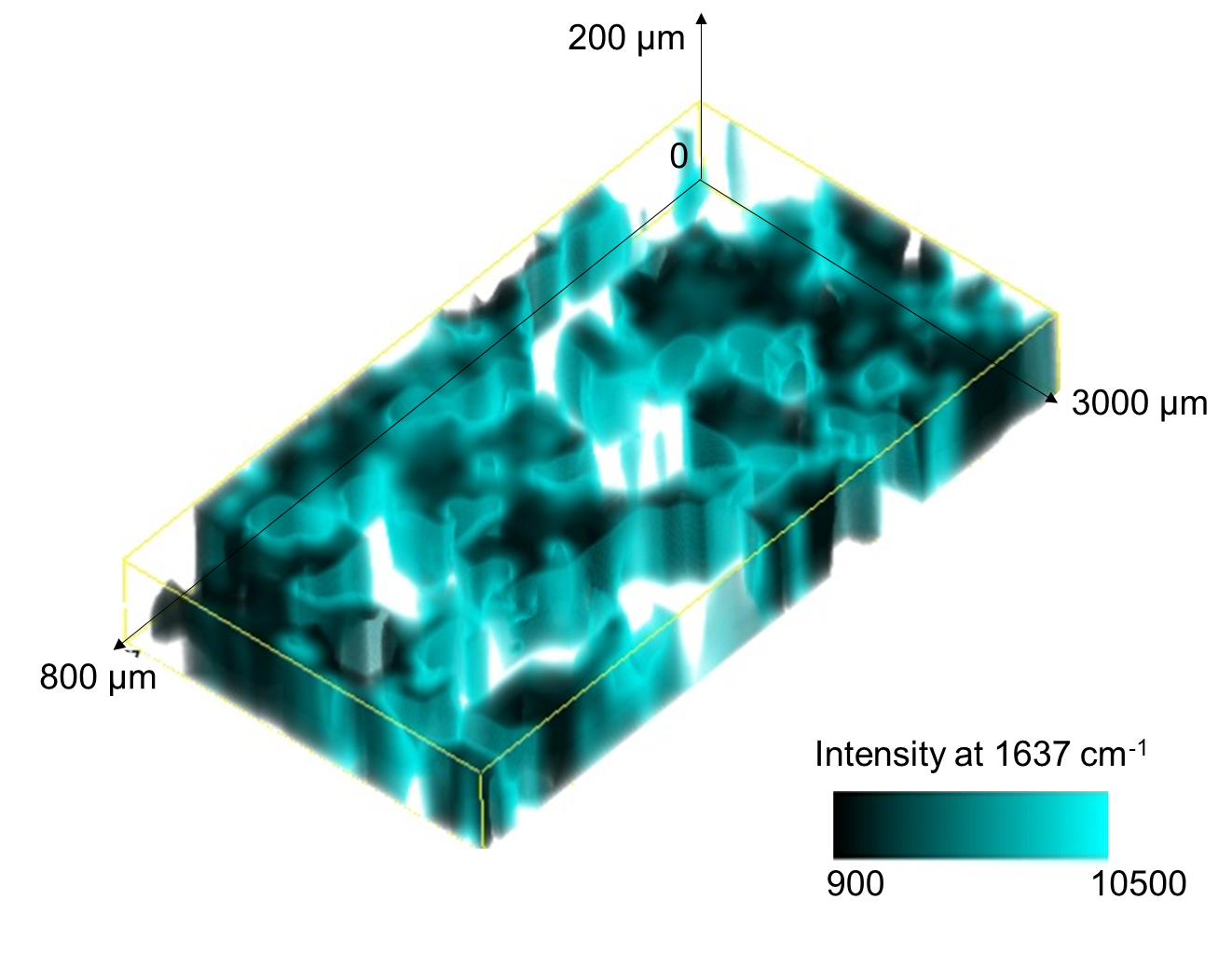


**Suppl. Fig. 13.** 3D SERS map of AgF@AgM@C10 after deposition of MB (584 × 584 × 60 μm^3^). The vibration at 1637 cm^-1^ is used to make the color scale.


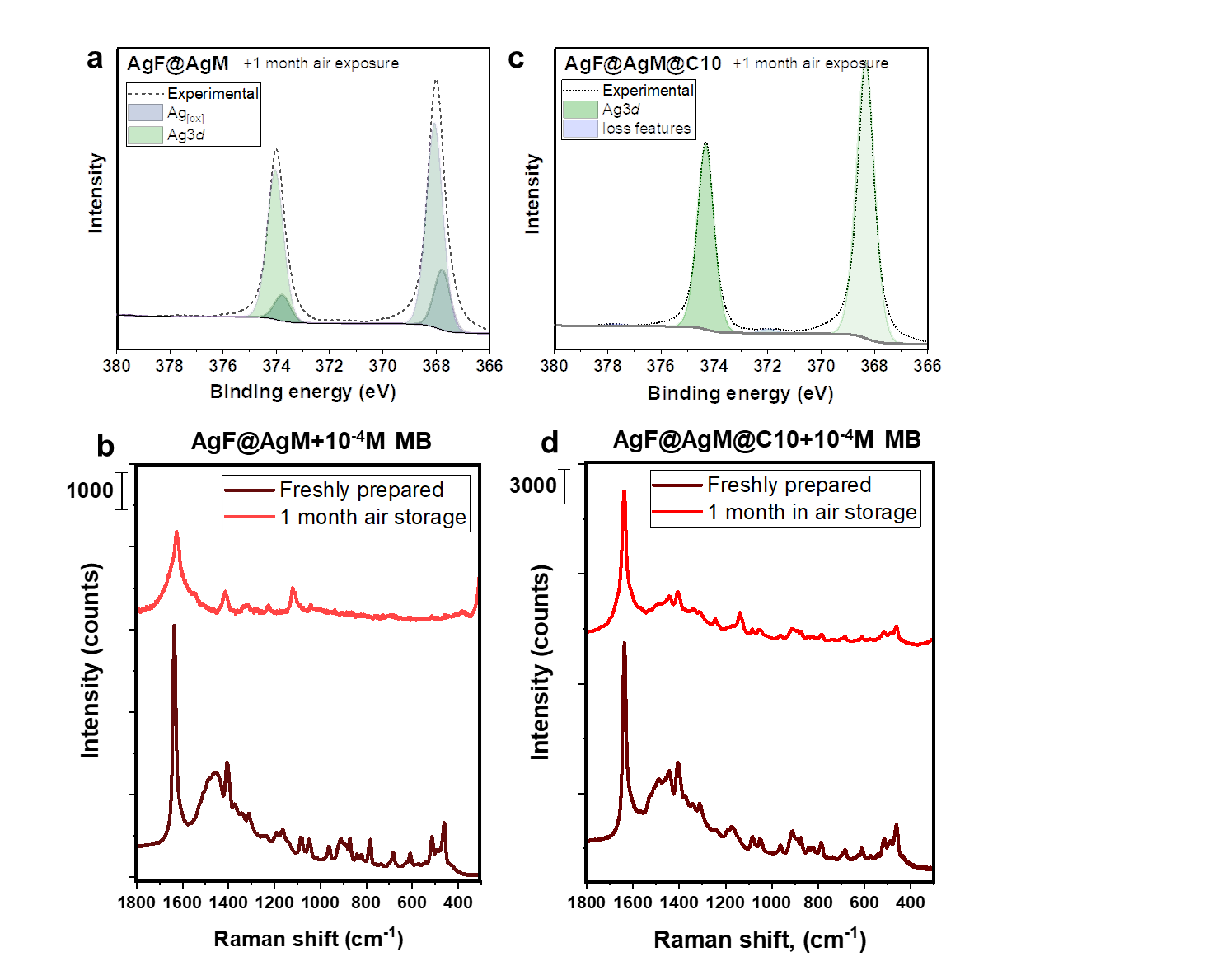


**Suppl. Fig. 14.** Stability tests for AgF@AgM@C10 and AgF@AgM towards oxidation in air. This data shows the protective role of 4-decylphenyl layer. Ag 3*d* XPS spectra and SERS spectra of MB (10^-4^ M) are collected after storage in air for 1 month (**a**, **b** AgF@AgM and **c**, **d** AgF@AgM@C10). XPS spectrum of AgF@AgM after storage in air shows that Ag 3*d*_5/2_ peak is deconvoluted to 368.1 eV and 367. 7 eV in contrast to a single peak before storage at 368.2 eV on Suppl. Fig. 5b. The shift to shorter energies indicates the formation of Ag_2_O.^10^ The Ag 3*d* XPS spectrum of AgF@AgM@C10 does not exhibit significant changes over time, consistent with a negligible decrease in plasmonic activity.


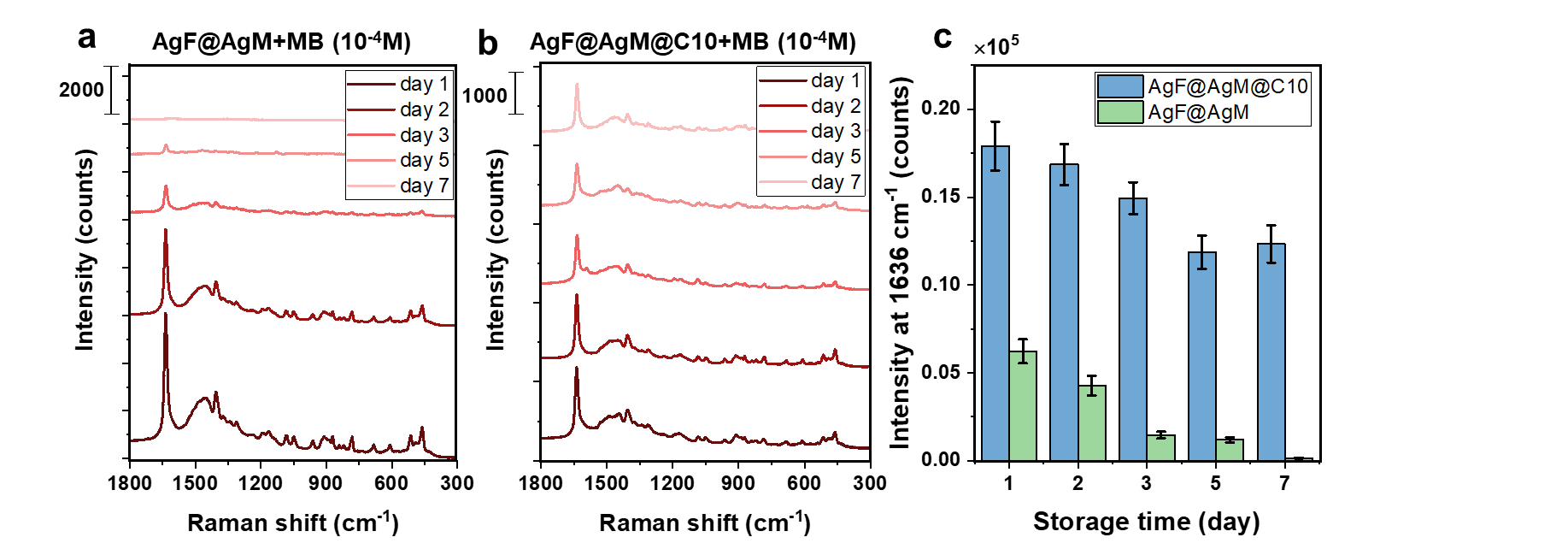


**Suppl. Fig. 15.** Stability tests for AgF@AgM@C10 and AgF@AgM towards oxidation in water. This data shows the protective role of 4-decylphenyl layer for the SERS enhancement.^10^ SERS spectra of MB (10^-4^ M) are collected from **a** AgF@AgM and **b** AgF@AgM@C10 after storage in water for 1,2,3,5,7 days. The decreased plasmonic activity **c** of AgF@AgM can be explained by oxidation. The mean value, calculated from three measurements on three samples (*N* = 3, *n* = 3), is presented.


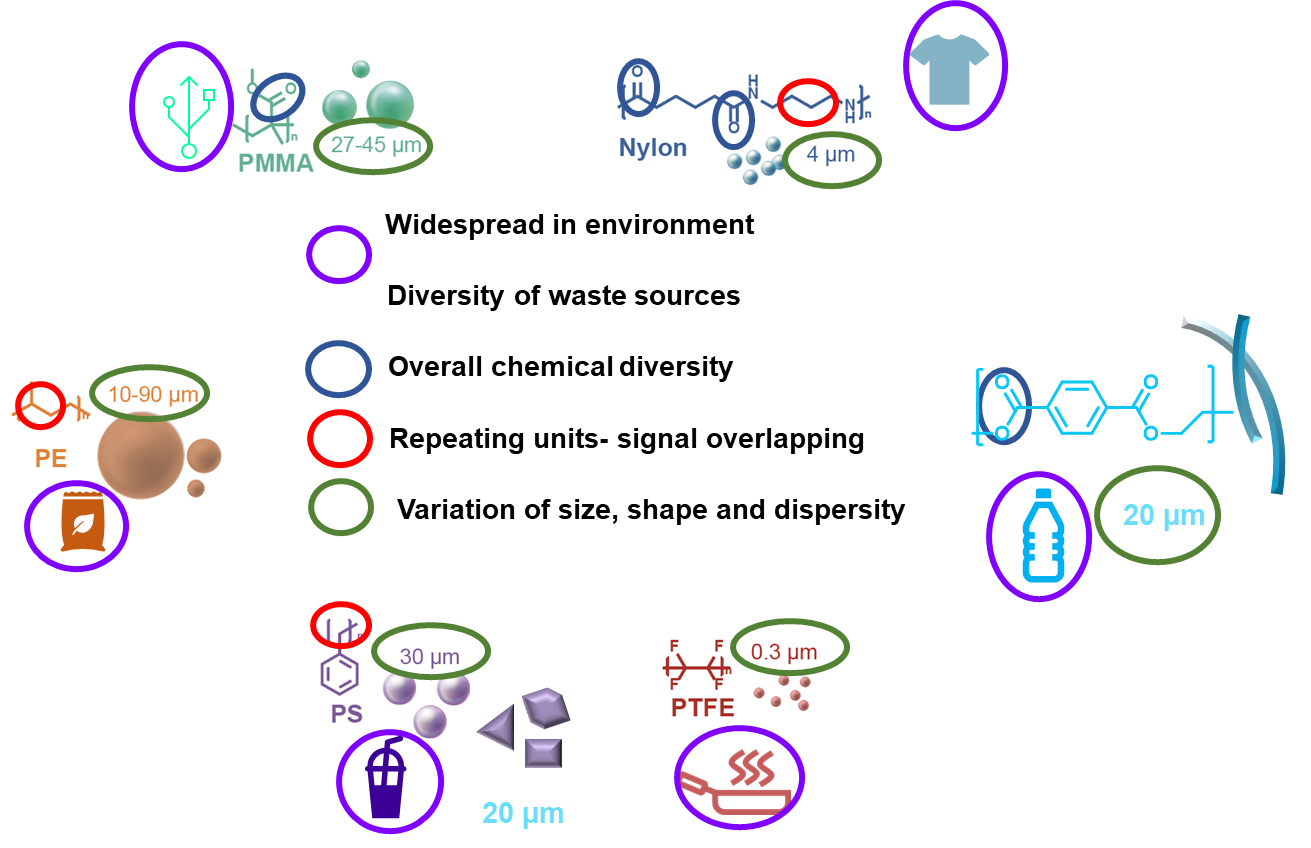


**Suppl. Fig. 16.** Explanation of the choice of microplastics (MPs). The scope of microplastics (MPs) can be explained by their widespread presence in the environment, the diversity of plastic waste sources, and the variation in the chemical structure of polymers, illustrated by selected examples of repeating units, size, and shape.

***Supplementary Note 3 to Suppl. Fig. 16.***

The choice of MPs is due to the following reasons:

**1) Diversity of plastic waste sources**

The choice of MPs is also aimed at covering different sources of MP to show the universality of the method in the detection of polymers originating from low-tech and high-tech industries. PET, PS, and PE are used to make single-use beverage bottles, packaging, and clothing. Simultaneously, PE and PS are commonly found in industrial waste associated with electronic equipment and energy production. PMMA waste is commonly associated with construction materials and electronics waste. Nylon and PET fibers are most commonly associated with textiles, although they are also incorporated in materials used by the fishing industry. Finally, PTFE is commonly used as dry and semi-solid lubricants (*i.e.*, powders/films and grease).

**2) Widespread in the environment (seawaters, waste and industrial waters, soil and sediments)**

MPs are chosen based on the relative abundance in the environment. Thus, across all types of plastic, PET, PS, PE, and Nylon are considered the most abundant sources of microplastic contamination in marine and freshwaters due to their widespread use in textiles, containers, construction materials, lubricants, etc.^11^ Despite the lower concentration of PMMA, the relative abundance is still high in the range of regions such as South China^12^ and South Japan^13^. In comparison with the most abundant microplastics, PFTE plays an important role as the polymer with relatively low content in the surrounding media demonstrating the sensitivity of proposed methods.

**3) Diversity in the chemical structure of polymers with selected examples of repeating units**

An MP detection method must be sensitive to polymers with wide structural diversity (*e.g.*, fluorinated groups, carboxylic groups, and aliphatic groups) while also being capable of discriminating between polymers with similar repeating structural units that would cause overlapping in the SERS spectra and hamper SERS-based identification methods.^14^ For example, Nylon, PMMA, and PET have carbonyl groups that generate vibrational bands with similar frequencies in Raman spectroscopy, as do alkyl groups in PS, PE, and Nylon. PTFE is introduced as an outlier because it has sufficient structural deviations and relatively low-intensity C-F and C-C vibration bands that are difficult to measure with SERS.

**4) Shape**

The shape of real MPs in the environment tends to be irregular because chemical and photochemical degradation is not well controlled. So, in this study, we initially measure polymers with a range of shapes. Then, we also test MPs with more irregular shapes by exposing the MPs to photo-Fenton degradation and then measuring them with SERS.

**5) Size**

The size of MPs in the environment is very diverse, from nanometers to hundreds of micrometers. Moreover, the dispersity of MPs is large due to the different sources. Therefore, in this study, we test MPs with a wide range of sizes from 300 nm to 90 µm, where the dispersity of PMMA, PE PS (fragments), and PET fibers is extremely low.


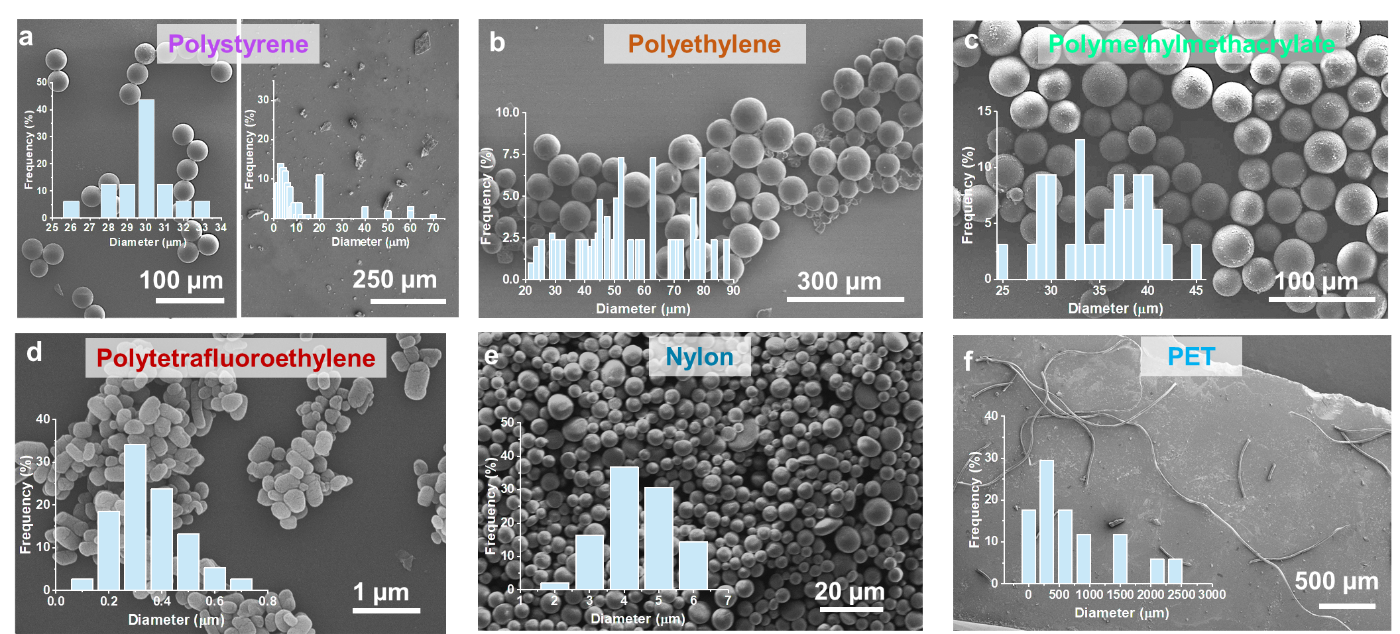


**Suppl. Fig. 17.** SEM images and size distribution histograms of MPs used in this study (**a** PS – beads and fragments, **b** PE, **c** PMMA, **d** PTFE, **e** Nylon, **f** PET (diameter of the fiber is 20±2 µm). The size distribution histograms show frequencies of MPs of different sizes found on SEM images of 3 samples of each type of MPs determined by ImageJ software.


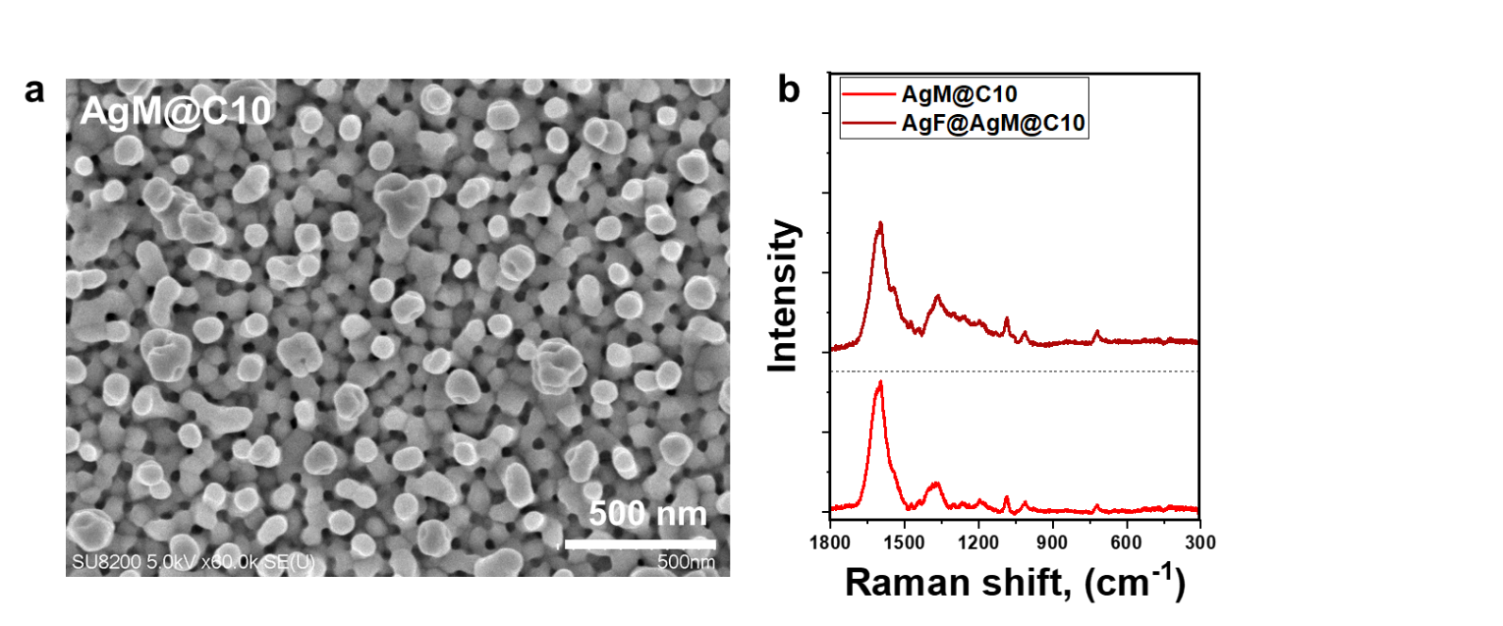


**Suppl. Fig. 18.** Characterization of AgM@C10 for further comparison with AgF@AgM@C10. **a** SEM image of AgM@C10 (deposition at -0.25 V for 1000 s on an Au-coated flat silicone substrate) showing the mesoporous structure with small particles on the surface and **b** SERS spectra in comparison with AgF@AgM@C10, confirming the successful functionalization due to similarity with AgF@AgM@C10.


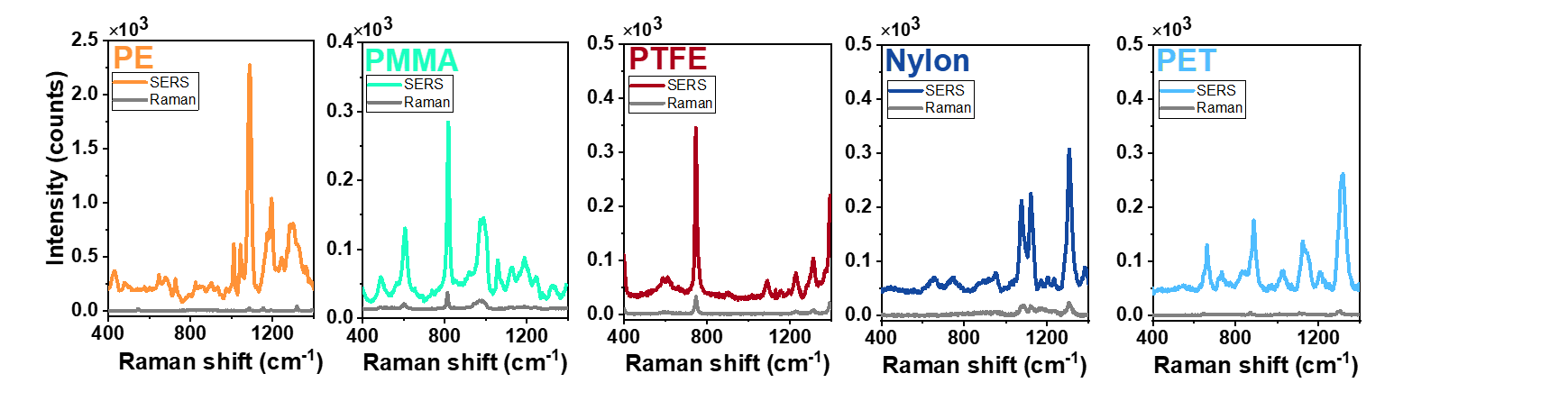


**Suppl. Fig. 19.** Comparison of EF for PE, PMMA, PTFE, Nylon, and PET obtained on AgF@AgM@C10 at 532 nm.


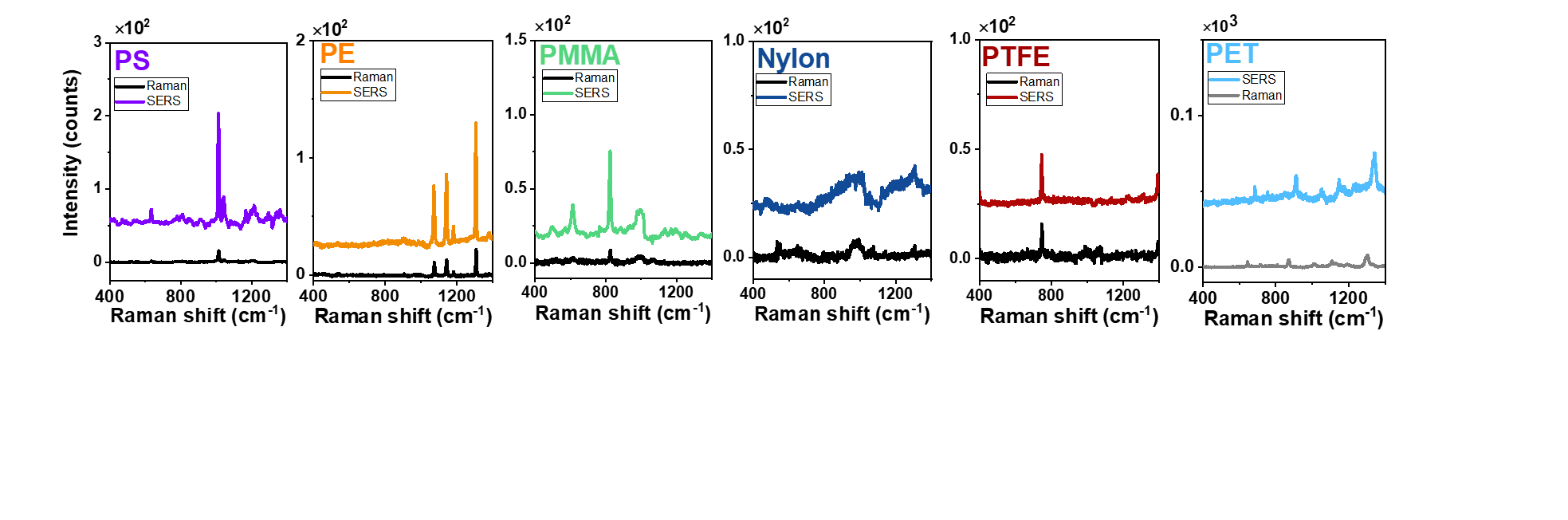


**Suppl. Fig. 20.** Comparison of EF for PS, PE, PMMA, Nylon, PTFE, and PET obtained on AgM@C10 at 532 nm.


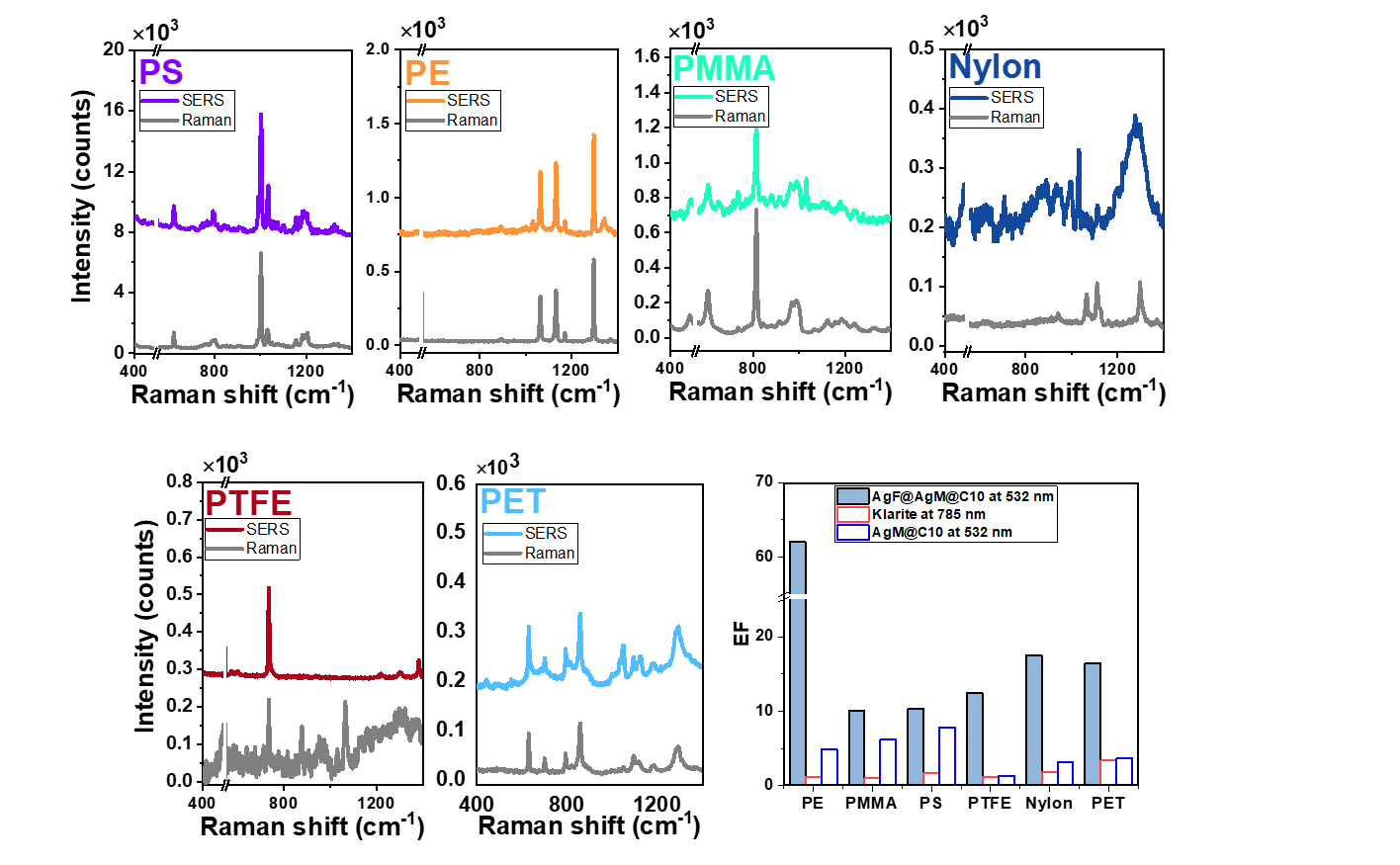


**Suppl. Fig. 21.** Comparison of EF for PS, PE, PMMA, Nylon, PTFE, and PET obtained on Klarite at 785 nm and comparison of EFs for MPs on AgF@AgM@C10, AgM@C10, and Klarite.


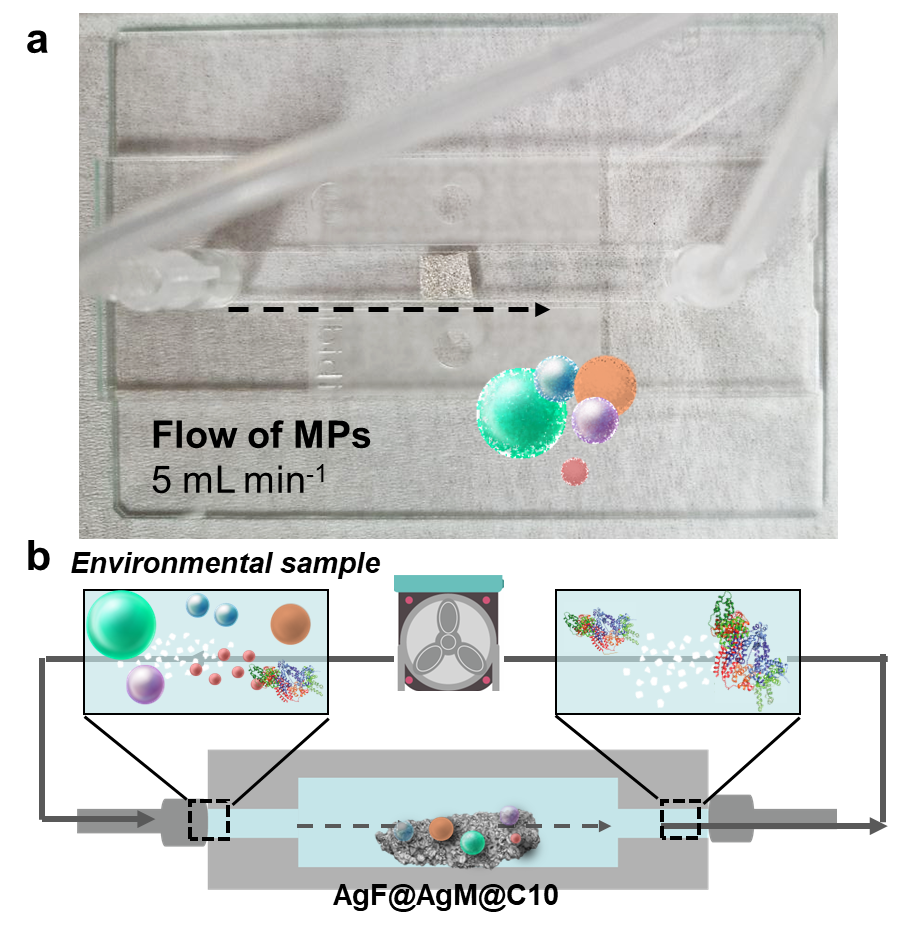


**Suppl. Fig. 22. a** Photograph of the AgF@AgM@C10 structure in the μ-Slide I Luer cell. A scheme shows how solutions are cycled through the sample. **b** Schematic representation demonstrating that MPs are trapped by AgF@AgM@C10, while other components partly flow through. The inserted images are extracted from previous literature sources.^15,16^


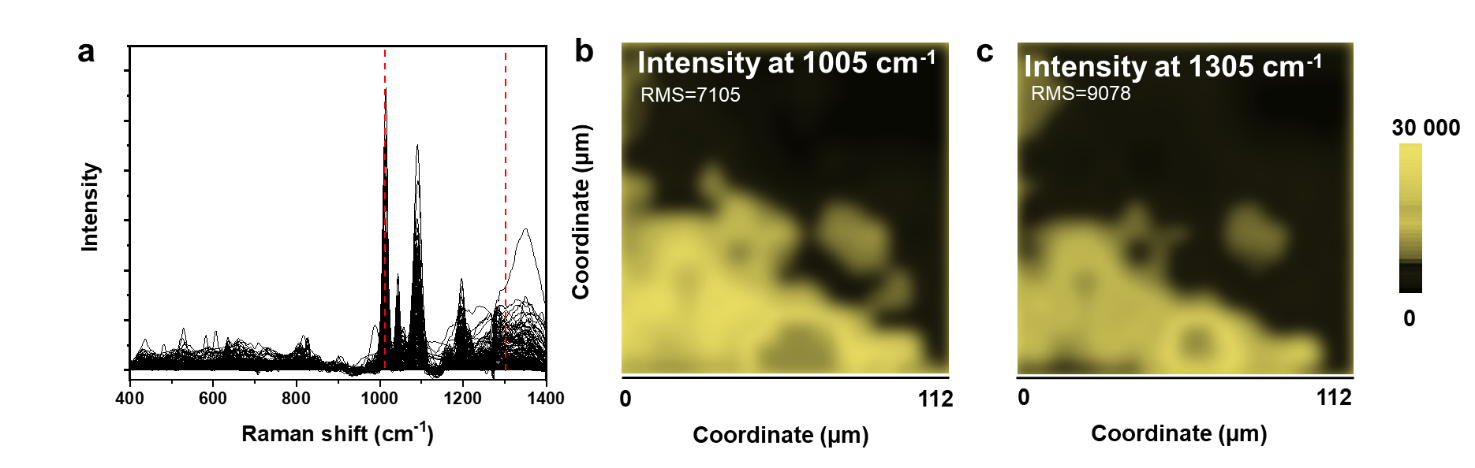


**Suppl. Fig. 23.** **a** SERS spectra and **b** 2D maps of AgF@AgM@C10 substrates exposed to PS and PE collected in a 0.1×0.1 mm^2^ area (0.5 µm step). The 2D maps visually depict the distribution of PS and PE through their respective peaks at 1005 cm^-1^ and 1305 cm^-1^.


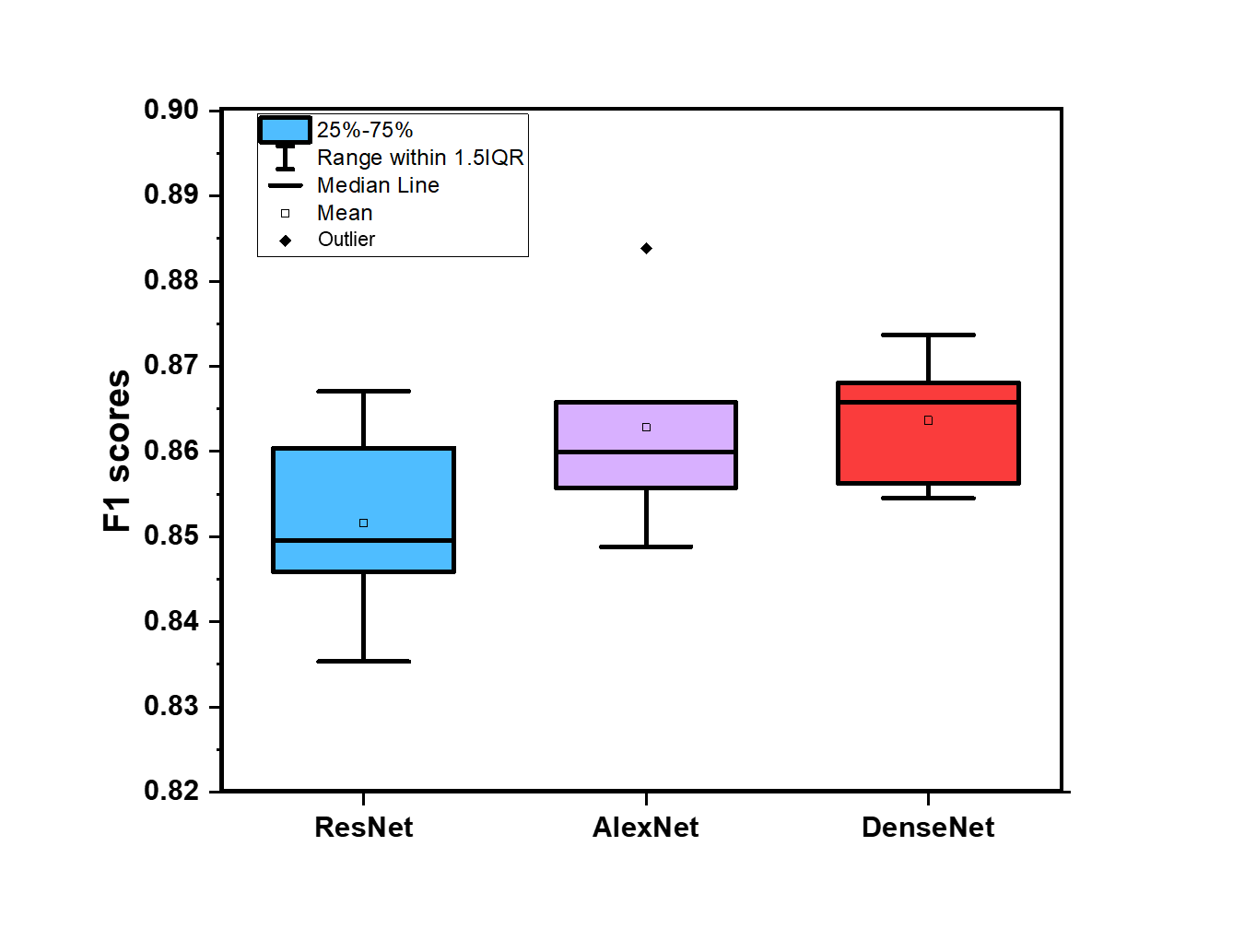


**Suppl. Fig. 24.** Comparison of the performance of various convolutional architectures (ResNet, AlexNet, DenseNet) in terms of F1 scores (*i.e.*, a balanced parameter between precision and recall) for MPs detection. All models perform similarly (ANOVA *p*-value = 0.39, *n*= 14).


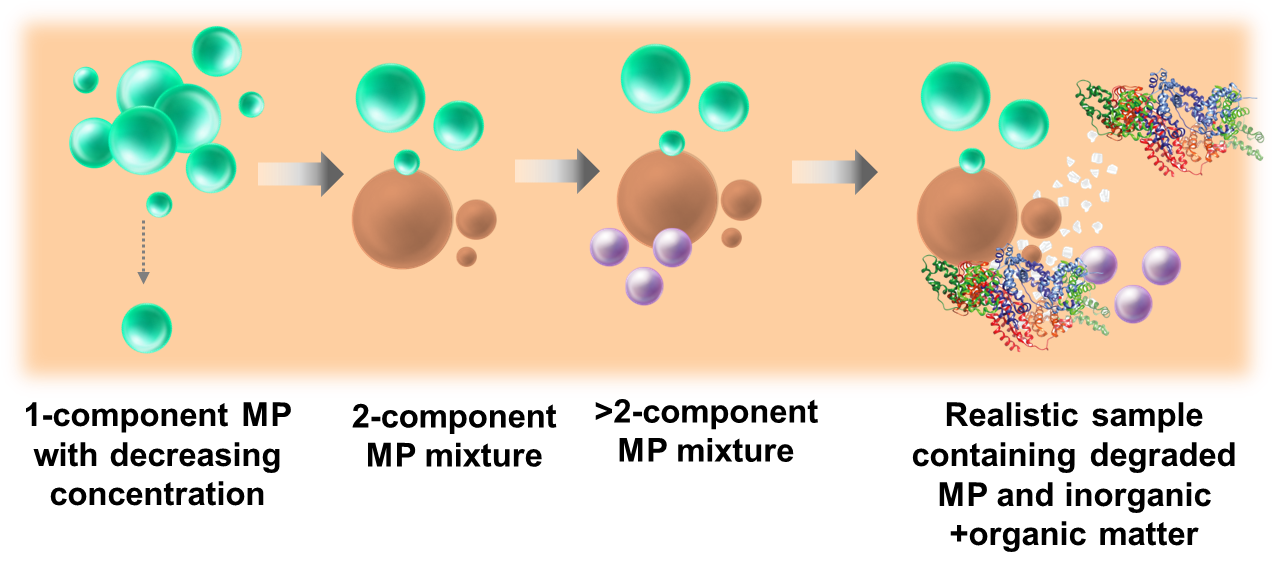


**Suppl. Fig. 25.** An illustration summarizing the types of sample types analyzed with SERS and subsequently subjected to the neural network for identification. The complexity of the samples is gradually increased. The inserted images are extracted from previous literature sources.^15,16^

.
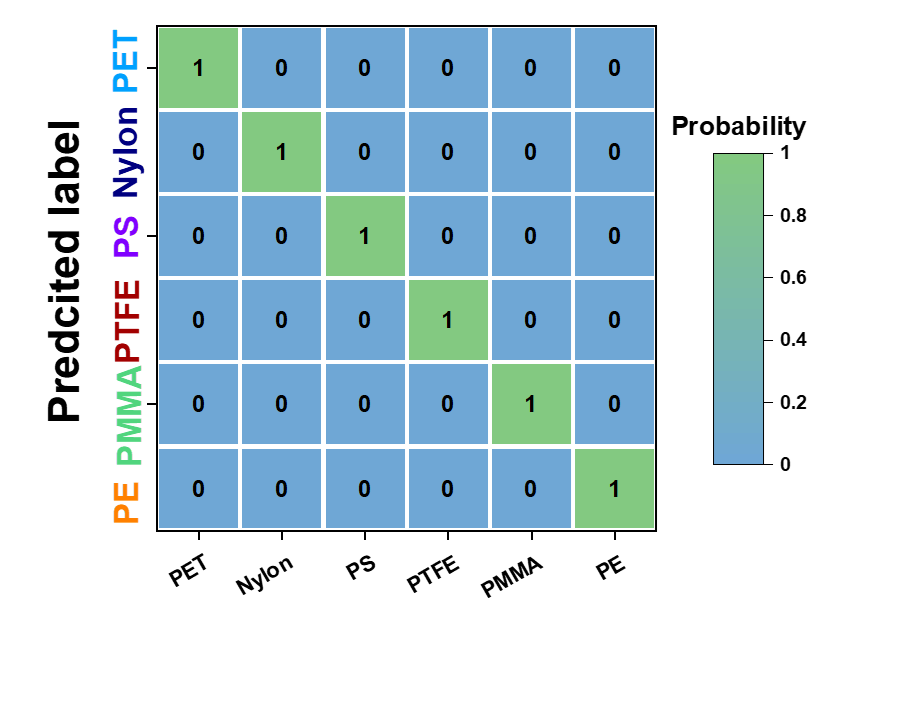


**Suppl. Fig. 26.** A confusion matrix describing the performance of SpecATNet with single-component MP samples (0.15 mg L^-1^).


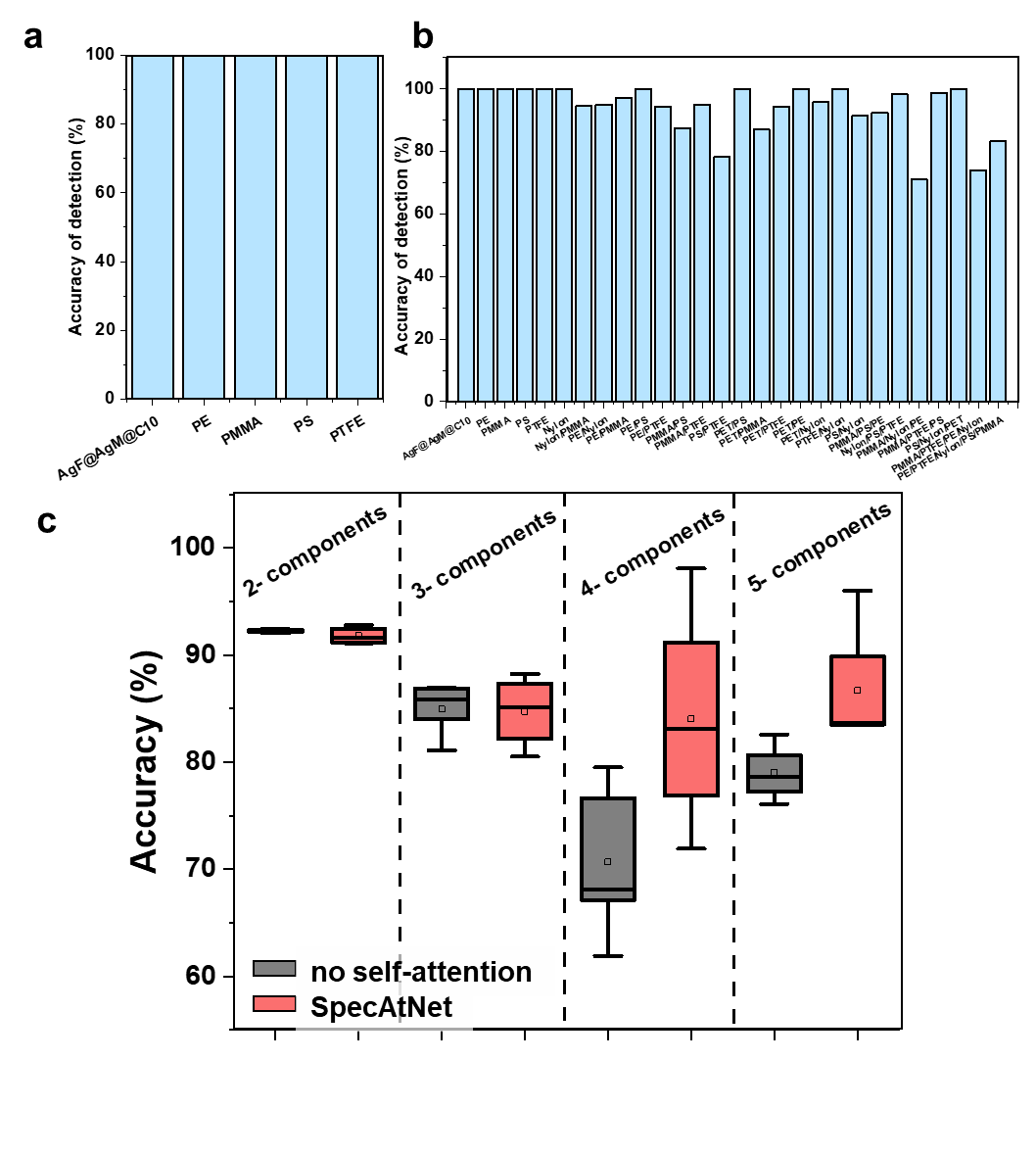


**Suppl. Fig. 27.** Accuracies of SpecATNet predictions for **a** single MP sample, **b** multicomponent samples, and **c** a comparison of the accuracy obtained from 2, 3, 4, and 5 component MPs mixtures by SpecATNet and analogous NN without the self-attention layer, with *p*-values of 0.99, 0.69, 6.9×10-3, and 1.66×10^-6^, respectively (independent samples one-sided T-test, *n* = 29). While for 2- and 3-mixtures, self-attention brings no benefits over simple averaging, it becomes statistically significant for 4- and 5-component mixtures.


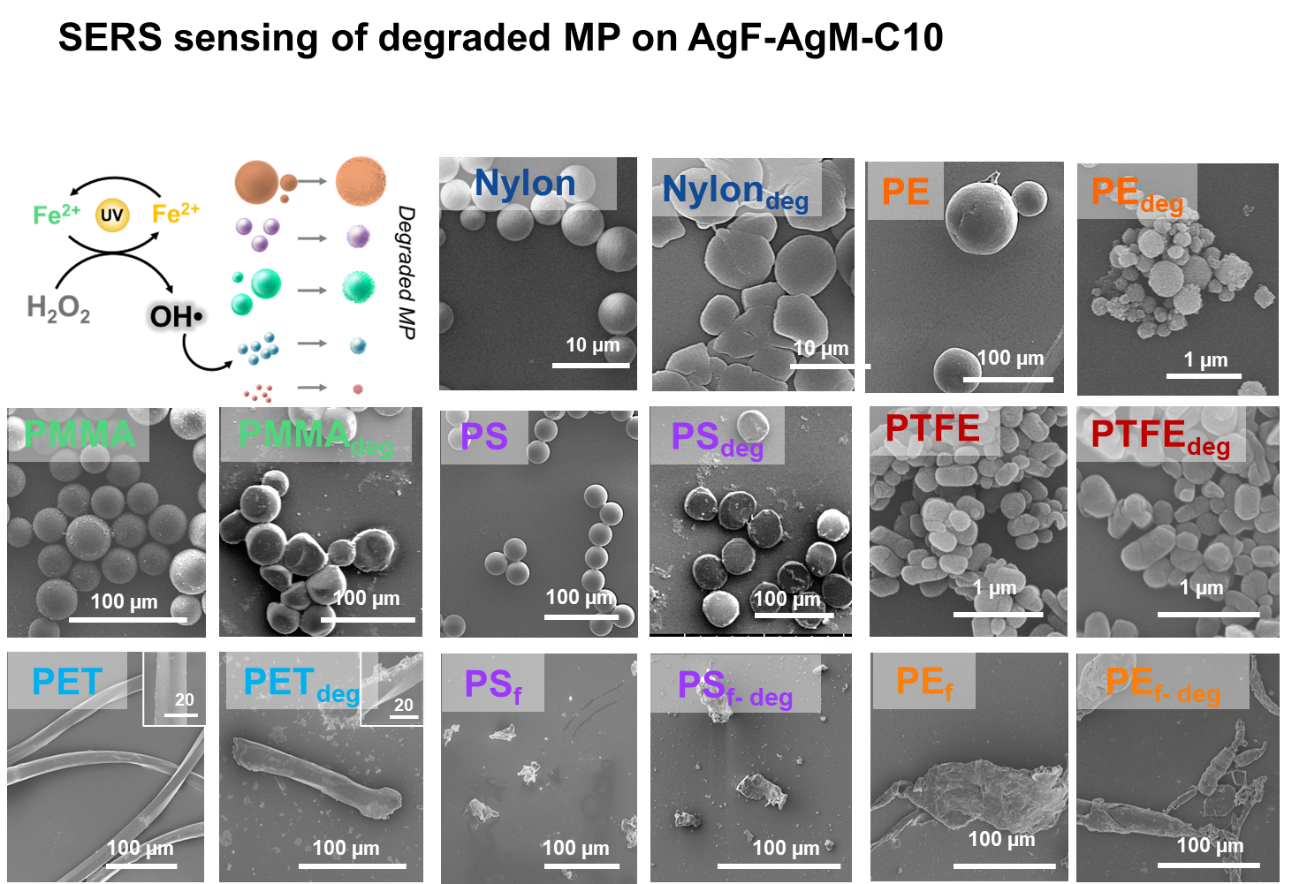


**Suppl. Fig. 28.** The photodegradation of different MPs *via* the Fenton reaction (*i.e.*, generation of reactive oxygen species by UV light exposure and exposure of FeCl_2_ and H_2_O_2_^17,18^). The mechanism and SEM images of each MP before and after photodegradation on the silicon substrates are presented here. The images labeled with “deg” indicate the samples after photodegradation.


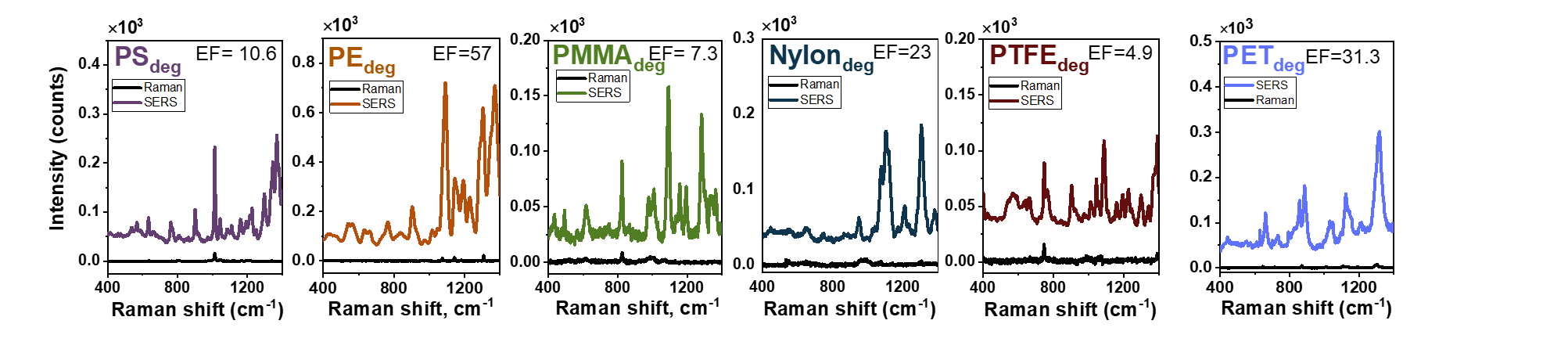


**Suppl. Fig. 29**. Comparison of EFs for degraded PS_deg_, PE_deg_, PMMA_deg_, Nylon_deg_, PTFE_deg_, and PET_deg_ on the glass.


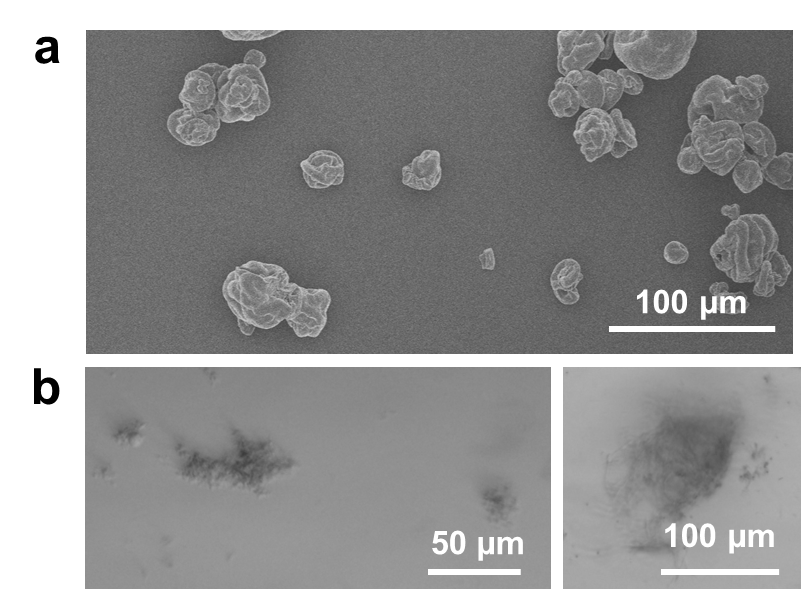


**Suppl. Fig. 30.** **a** SEM images of dried algae and **b** microscopical images of grown algae in Guillard’s (F/2) Marine Water.


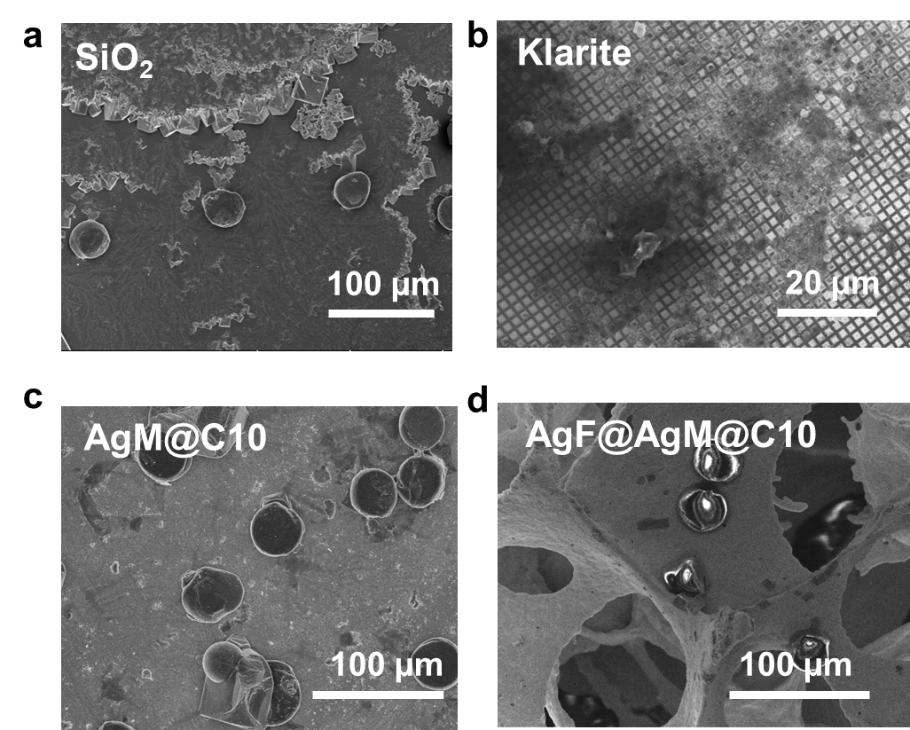


**Suppl. Fig. 31.** Examining the antifouling properties of different substrates. SEM images of the environmental sample (PE_deg_/PS_deg_+BSA+NaCl) deposited on **a** SiO_2_, **b** Klatite, **c** AgM@C10, and **d** AgF@AgM@C10. Only MP samples are observed inside the pores by circulating in a flow mode.


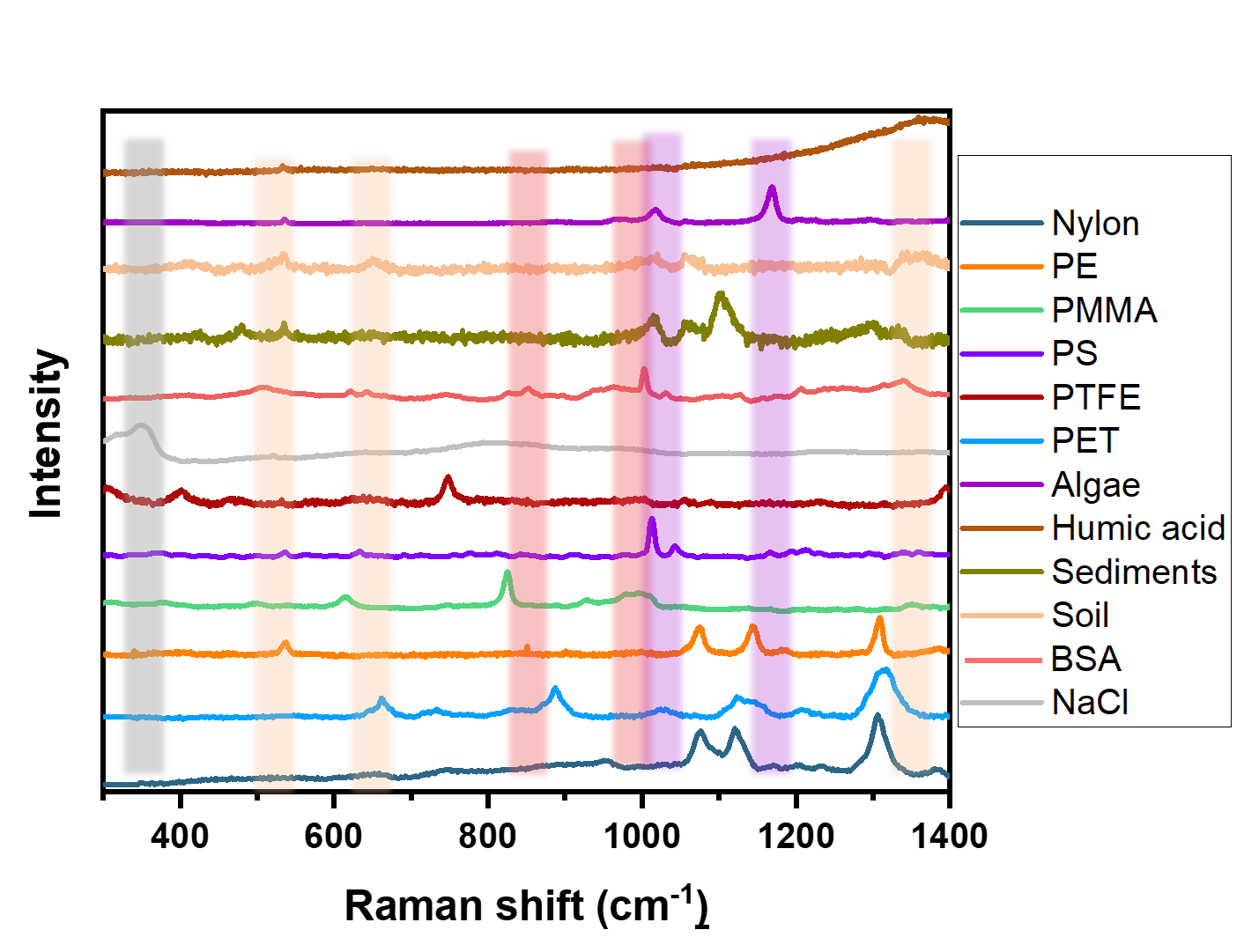


**Suppl. Fig. 32.** Raman spectra of algae, humic acid, sediments, soil, BSA, and NaCl in comparison with 6 types of MP. The peaks of the matrix overlap with some MPs and can interfere with their detection with signature peaks of MP, for example, at 510-535 and 1310-1340 cm^-1^


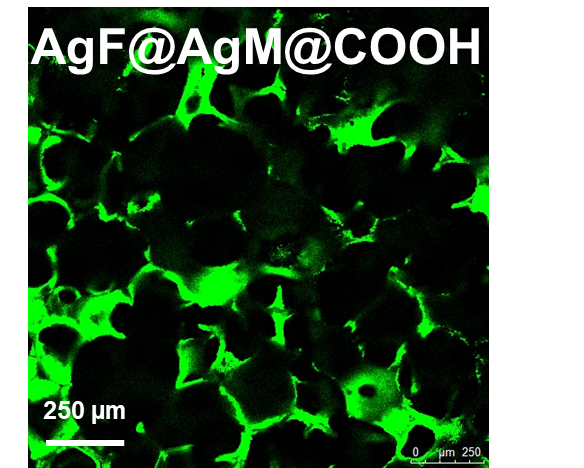


**Suppl. Fig. 33.** Fluorescence microscopy image of AgF@AgM@COOH following protein adhesion tests using fluorescein-conjugated BSA (BSA-FITC).


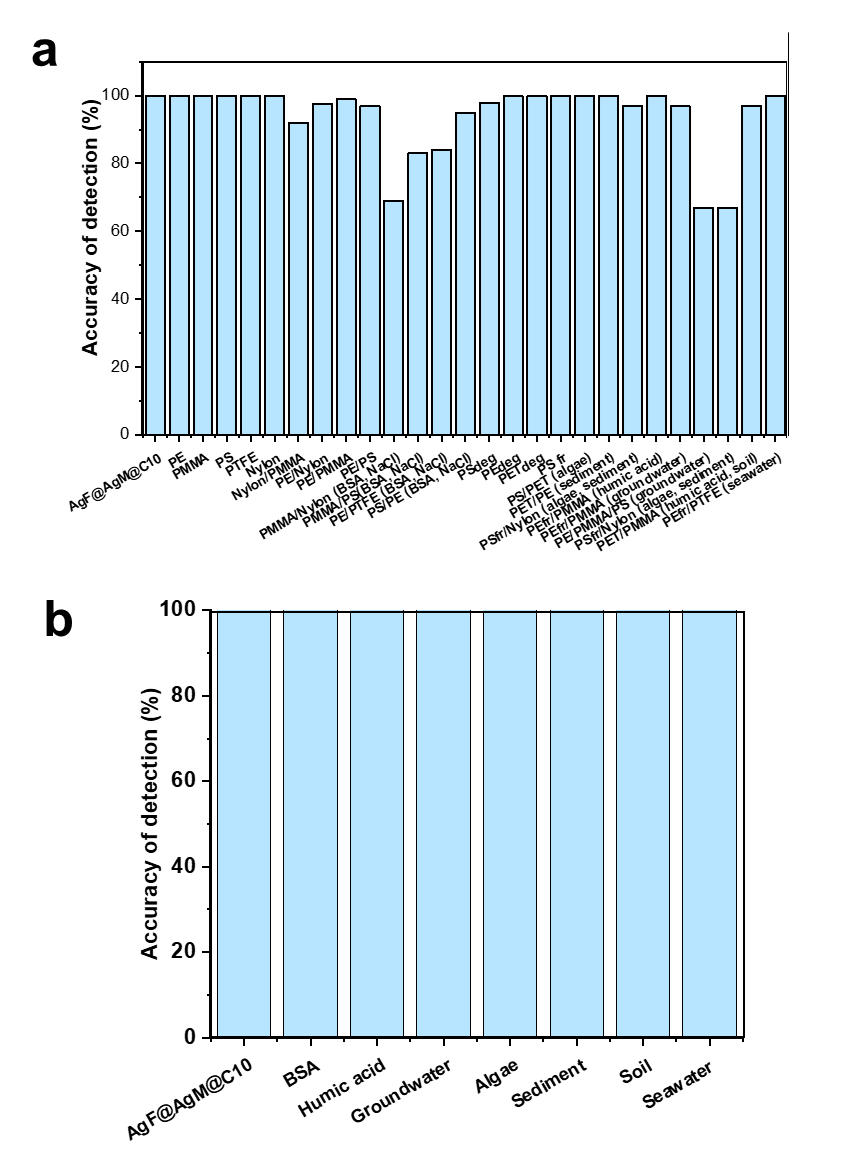


**Suppl. Fig. 34. a** Accuracies of SpecATNet prediction for multi-component samples in complex matrices (PS_fr_ corresponds to PS fragments) and **b** matrices without MPs.


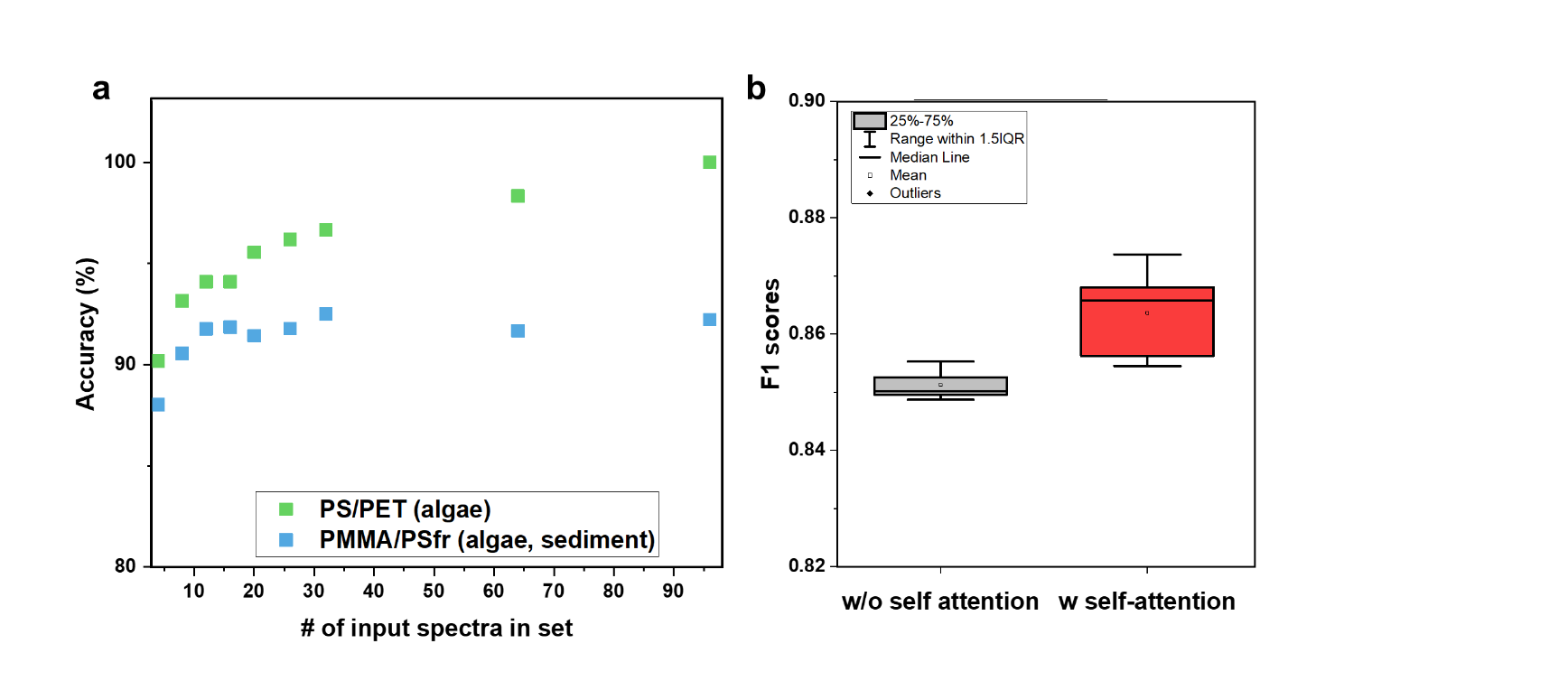


**Suppl. Fig. 35.** **a** Dependence of accuracy on the number of spectra in input spectral set for PS/PET and PMMA/PS_fr_ in complex matrices (PS_fr_ corresponds to PS fragments) and **b** comparison of the F1 score of SpecATNet versus a NN without using the self-attention. The model with self-attention demonstrates a statistically significant increase in prediction quality (*p*-value = 0.013, independent samples one-sided T-test, *n* = 29, multiple convolutional part architectures included).


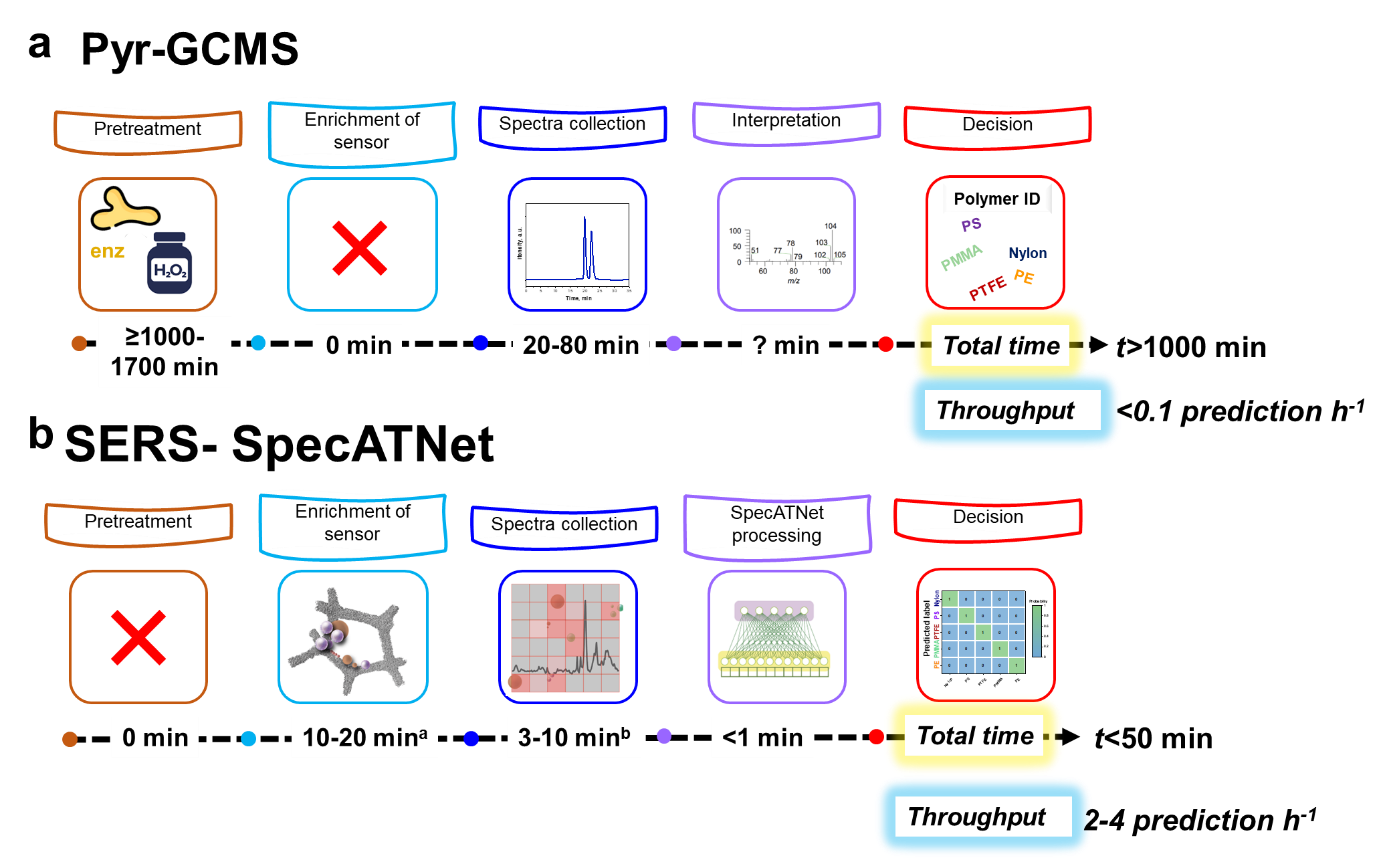


**Suppl. Fig. 36.** Timeline of **a** Pyr-GCMS and **b** SERS-SpecATNet-assisted analysis of environmental MPs samples.

***Supplementary Note 4 to Suppl. Fig. 36:***

Pyr-GCMS is a powerful technique, but it requires pretreatment by filtration, digestion, or chemical extraction because the environmental matrix produces interfering signals (Suppl. Table 1). The interpretation of mass spectra of MP is already challenging and requires chemical derivatization, and interfering signals make analysis more difficult. From the literature survey,^9,20^ the pretreatment takes more than 12 hrs, which is the bottleneck in sensor throughput. The Pyr-GCMS data analysis has not been automized by ML probably due to differences in mass spectra depending on sample type. Therefore, it was impossible to evaluate this step. To sum up, sensor throughput was tentatively evaluated as 0.1 prediction h^-1^.

SERS-SpecATNet needs 20 min to enrich the sensor by the flowing water sample through porous AgF@AgM@C10, this step can be performed simultaneously on multiple samples to accelerate the process. Sensor enrichment is followed by collecting the SERS dataset from the sample, where 20 spectra are minimum required taking 3 min (10 min for 60 spectra). The processing of the dataset takes less than a minute, giving 2-4 predictions h^-1^, which is at least ×20 times faster than Pyr -GCMS and more suitable for a practical setting.


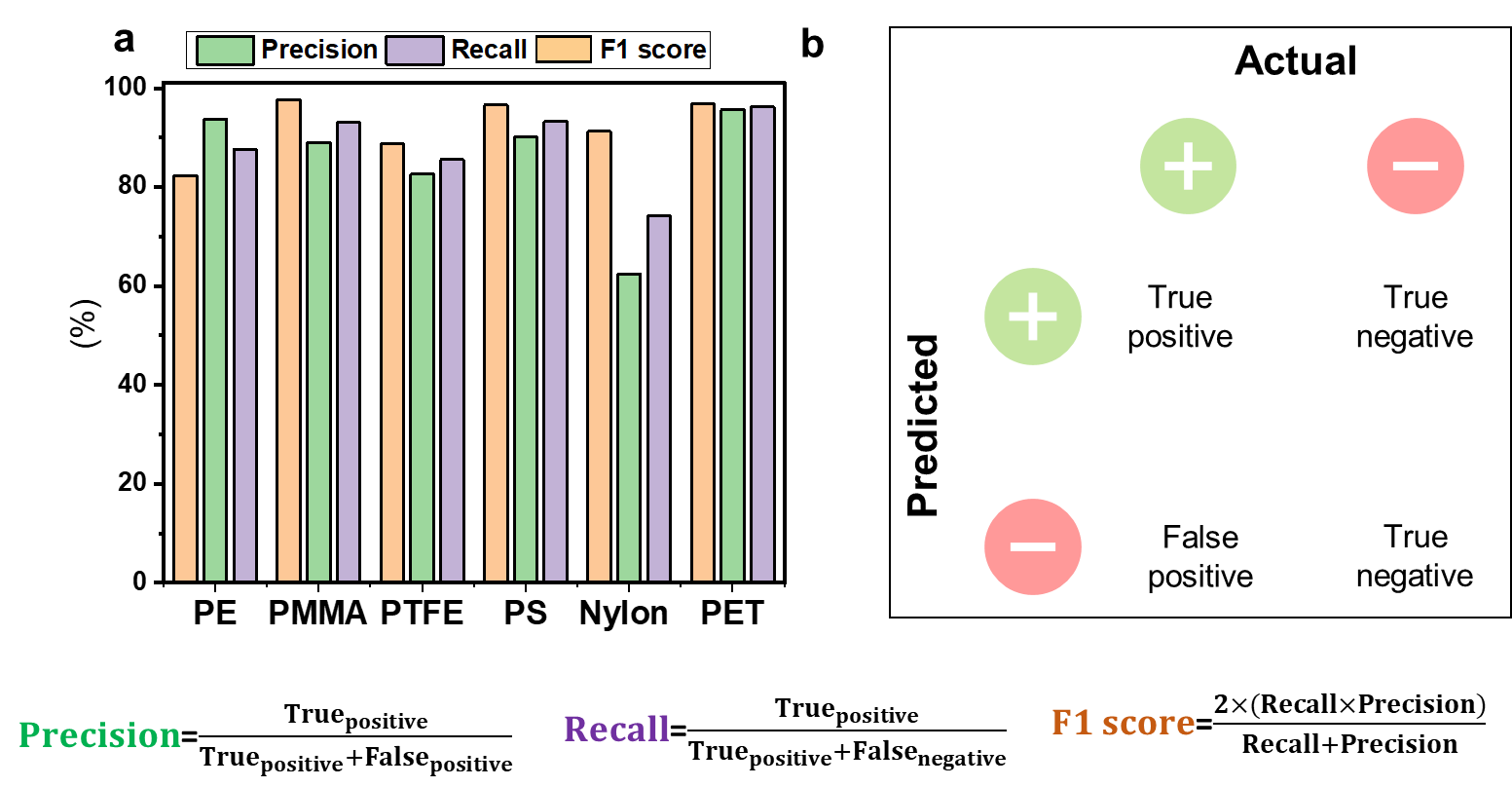


**Suppl. Fig. 37.** **a** Overview of the precision, recall and F1 score based on all the samples analyzed in this study on AgF@AgM@C10 and **b** schematic explanation of true positive true negative false positive, and false negative.


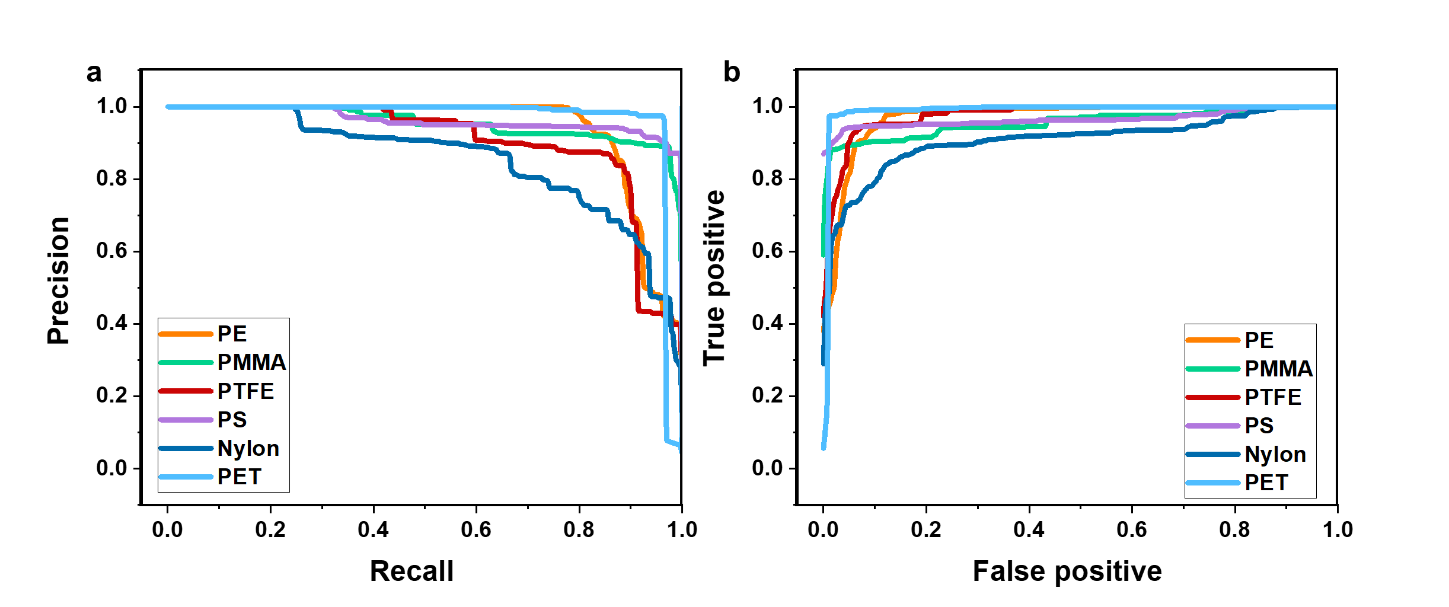


**Suppl. Fig. 38**. **a** Precision-recall curve and **b** receiver operating characteristic curve for six types of MPs.


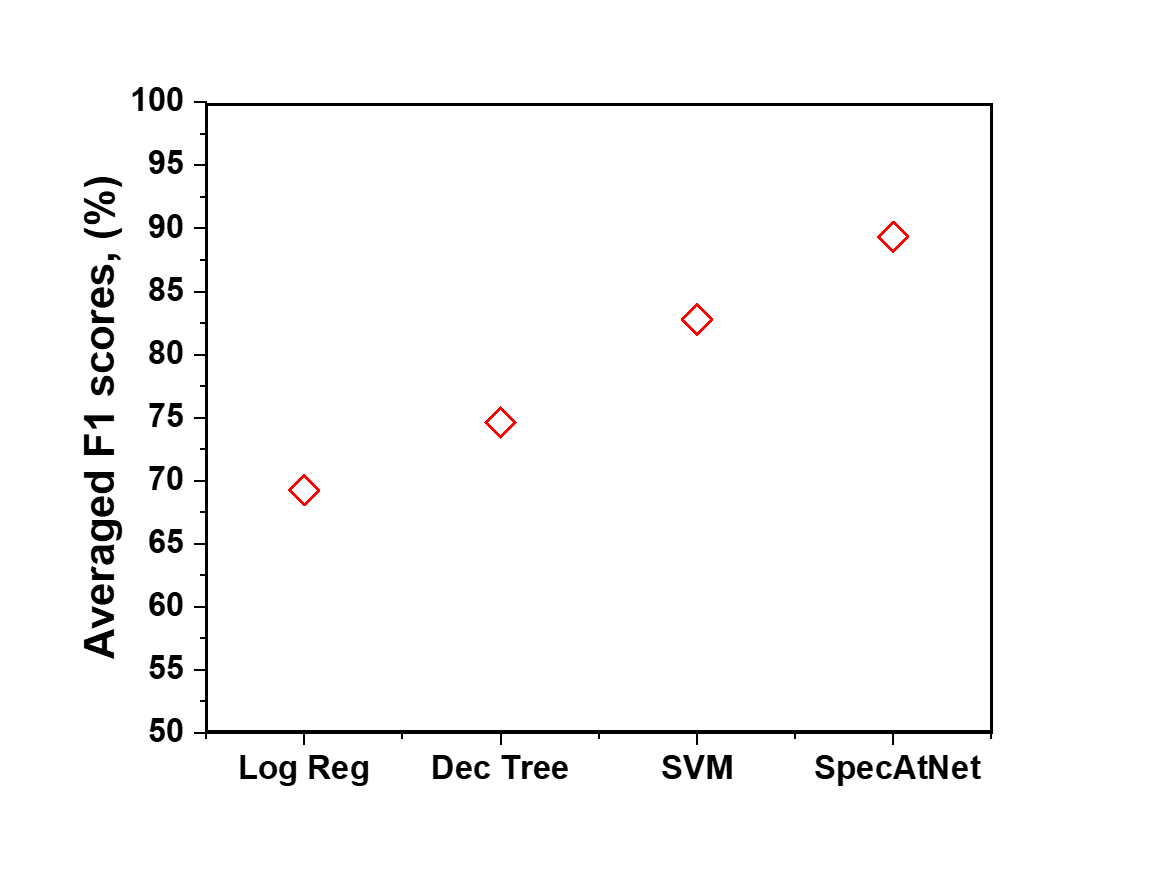


**Suppl. Fig. 39.** Comparison of F1 scores obtained from logical regression model (Log Reg), decision tree model (Dec Tree), support vector machine (SVM), and SpecATNet. The diamonds represent the average F1 score for 6 MP types.


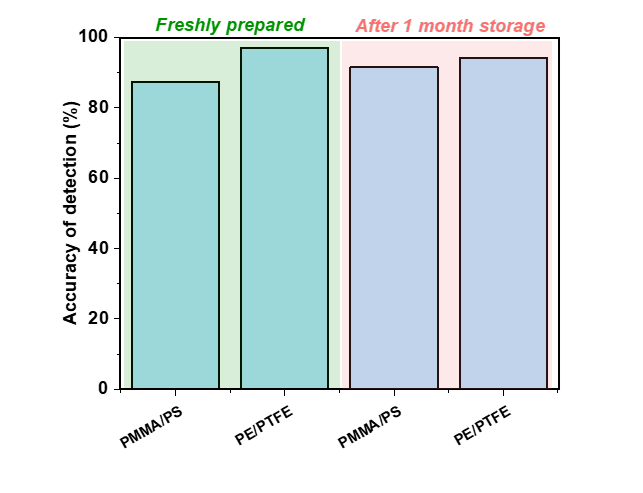


**Suppl. Fig. 40.** Comparison of freshly prepared and after 1 month air storage AgF@AgM@C10 performance for sensing MPs mixtures.


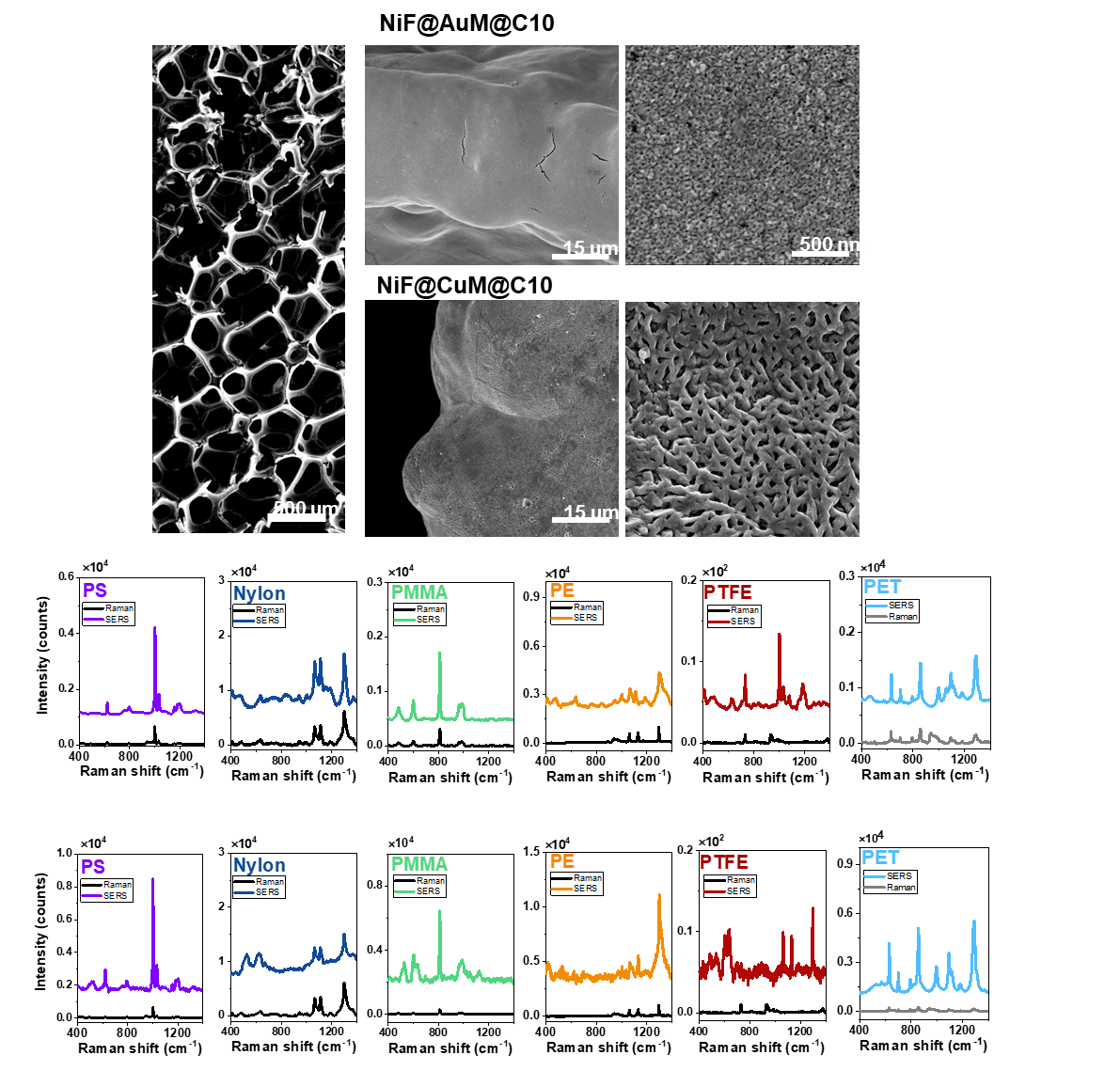


**Suppl. Fig. 41.** Possible extension to other mesoporous Au and Cu depositions on Ni foam (NiF@AuM@C10 and NiF@CuM@C10) to reduce the price and preliminary data on SERS sensing of MPs on NiF@AuM@C10 and NiF@CuM@C10 at 785 nm.

***Supplementary Note 5 to Suppl. Fig. 41.***

The strategy of creating optimal macroporous networks with electrodepositing mesoporous metal films with different metals and pore sizes can be widened for SERS sensing of large macromolecules. The benefits of this strategy are i) the possibility to change average macropore diameter to match the macro analyte size, ii) the possibility to change mesopore size and metal to regulate optical properties, iii) creation of 3D plasmonic field exciting analyte from different sides. We estimate that a 1 cm^2^ of AgF@AgM@C10 substrate (1 mm thick) would cost ≈4 $ without optimizing for economies of scale. For reference, Ag nanopillar substrates produced by Silmeco cost 50 to 77 $, depending on the number of units purchased.^19^

Our lab has demonstrated the ability to deposit numerous kinds of mesoporous metals (*e.g.*, Au, Ag, Cu, Pt, Pd, Ir, Ru, Rh, *etc*.) on different types of substrates.^20^ As a proof of concept to optimize the cost of our SERS microplastic sensors, we electrodeposited Au and Cu on nickel (Ni) foam after pretreating the Ni foam with acid. Using the inexpensive Ni substrate decreased the cost of the sensor to ≈1.2 $. Using Cu precursor and Ni substrates can decrease the cost of the sensor to <0.5 $. Both sensors are capable of measuring the MPs used in this manuscript even though the conditions have not been optimized (Suppl. Fig. 41). Mesoporous metals can be deposited onto conductive polymers, or the method can be modified to deposit metal via electroless deposition. In that case, the cost of these sensors should be minuscule (<≈0.4 $) even in the most resource-limited environment. For a complete comparison of cost, see Suppl. Table 8.

**Suppl. Table 1.** Comparative table of visual, mass, and optical spectroscopic strategies for MPs analysis.

| **Parameters** | **Visual** | | **Mass** | | **Optical spectroscopy** | |
| --- | --- | --- | --- | --- | --- | --- |
| Detection tools | Naked eye, digital camera, microscopy (light, dissecting, confocal, scanning electron, atomic force) | | Pyr-GCMS, TGA-MS/DSC | | FTIR, Raman, SERS | |
|  | **General** | **Example** | **General** | **Example** | **General** | **Example** |
| Chemical analysis | No | - | Yes | PE, PP, PS, PET, PVC, PC, PMMA^21^;  PET ^22^ | Yes | CA, PS, PE, PP, PA ^23^;  PVC, PU, PS, PP, PE, PMMA^24,25^ |
| Detection of MP mixture | No (only visual) | ^26,27^ | Yes | ^21^ | Only one example | ^28^ |
| Size range | Yes | Digital camera – 1 mm ^26^;  Fluorescent^29^ – 100 µm | No | single particles measurement  50 µm^30^;  0.1 µm ^21^ | Yes | FTIR – >10-20 µm  Raman- >1 µm  SERS >360 nm^31^ |
| Environmental sample analysis | Yes | Marine sample^26^;  Marine sediments^32^ | Yes | cellulose, pine wood, wool, and cotton^33^; sediment sample^34^ | Yes | Marine sample  ^24,28^  Wastewater^35^ |
| Pretreatment | Required | density separation, staining ^26^; sieving, manual inspection^36^;  chemical digestion^32,37^;  extraction by organic solvent^38^ | Required | chemical and enzymatic digestion, derivatization^33,34^ | Required | Filtering, centrifugation, H_2_O_2_, NaI^39^  Manual selection^28^ |
| Portability | Yes (large MP) | Digital camera – 1 mm^26^ | No | ^40^ | Yes | smartphone-based Raman analyzer^41^;  portable Raman spectrometer^42^ |
| Operator qualification | Low | ^26^ | High | ^21,30^ | Moderate-Low | ^41,^ ^42^ |
| Price of analysis per sample | Cheap-moderate | Camera, common microscopes – cheap;  Other microscopies - moderate | High | ≈1000 €^43^ | Cheap-moderate | ≈300 €^44^ |
| Price of equipment |  | From ≈1000 € |  | From ≈100 000 € |  | From ≈3700 € |
| Combination with NNs | Yes | ^26,27^ | No | - | Yes | ^23–25,45^ |

**Suppl. Table 2.** Assigning bond vibrations to the Raman and SERS spectra of ADT-C_10_ and AgF@AgM@C10.^46^

| **ADT-C_10_** | | **AgF@AgM@C_10_** | |
| --- | --- | --- | --- |
| Raman shift  (cm^-1^) | Assignment | Raman shift  (cm^-1^) | Assignment |
| 2290 | N≡N stretching |  |  |
| 1588 | ring C=C stretching vibrations | 1574 | ring C=C stretching vibrations |
| 1440 | asym C-H deformation vibration | 1385 | asym C-H deformation vibration |
| 1300 | CH3 sym bending vibration | 1340 | asym C-H deformation vibration |
| 1213 | =C-H in-plane deformation vibrations | 1246 | =C-H in-plane deformation vibrations |
| 1163 | C-C skeletal vibration, =C-H in-plane deformation vibrations | 1153 | C-C skeletal vibration, =C-H in-plane deformation vibrations |
| 1133 | C-C skeletal vibration | 1101 | C-C skeletal vibration |
| 1096 | C-C skeletal vibration, =C-H in-plane deformation vibrations |  |  |
| 1084 | CCC stretching |  |  |
| 1042 | C-C skeletal vibration, Aromatic =C-H in-plane deformation vibrations | 1022 | C-C skeletal vibration, Aromatic =C-H in-plane deformation vibrations |
| 823 | C-C skeletal vibration | 980 | C-C skeletal vibration |
| 805 | =C-H out-of-plane deformation vibrations | 809 | =C-H out-of-plane deformation vibrations |
| 717 | =C-H out-of-plane deformation vibrations | 715 | =C-H out-of-plane deformation vibrations |
| 646 | Aromatic ring deformation vibration | 620 | Aromatic ring deformation vibration |
|  |  | 421 | Ag-C |

**Suppl. Table 3**. Assigning bond vibrations to the Raman spectra of PS, PE, PMMA, Nylon, and PTFE.^46^

| **Microplastics** | **Raman shift (cm^-1^)** | **Assignment** |
| --- | --- | --- |
| PS | 1209-1185 | C-C stretch |
|  | 1156 | C-C stretch |
|  | 1030 | C-H in plane deformation |
|  | 1005 | Ring breather mode |
|  | 795 | C-H out of plane deformation |
|  | 618 | Ring deformation mode |
| PE | 1300-1305 | CH_2_ twisting |
|  | 1170 | C-C stretch |
|  | 1130 | C-C stretch |
|  | 1063 | C-H out of plane deformation |
| PMMA | 991 | O-CH rocking vibrations |
|  | 970 | CH_3_ rocking |
|  | 815 | C-O-C stretch |
|  | 602 | C-O-C bending |
|  | 483 | C-O-C bending |
| Nylon | 1300 | CH_2_ twisting |
|  | 1110 | C-C stretch |
|  | 1064 | C-C stretch |
|  | 940 | C-CO stretch |
| PTFE | 1385 | C-C stretching |
|  | 1302 | C-C stretching |
|  | 1220 | antisymmetric CF_2_ stretching |
|  | 733 | symmetric CF_2_ stretching |
| PET | 1316 | C-H in plane bending |
|  | 1120-1146 | C-C stretch, |
|  | 1027 | C-O stretch |
|  | 885 | C-H out plane deformation |
|  | 734 | C-H out plane deformation |
|  | 663 | C-O-H bending |

**Suppl. Table 4.** Conversion of MP concentrations used in this study.

|  | PS | PMMA | PE | Nylon | PTFE |
| --- | --- | --- | --- | --- | --- |
| mg L^-1^ | 0.15 | 0.15 | 0.15 | 0.15 | 0.15 |
| MPs L^-1^ | 1.06×10^4^ | 5.5×10^4^ | 1.9×10^4^ | 2.9×10^6^ | 2.9×10^9^ |

**Suppl. Table 5.** Comparison of different approaches for identification of the chemical structure of MPs.

| **Method** | **Substrate** | **MP (Size)** | **Mixture detection** | **Source (pretreatment)** | **ML method** | **Accuracy (%)** | **Ref** |
| --- | --- | --- | --- | --- | --- | --- | --- |
| SERS | Klarite | PS, PMMA (360 nm~5 μm) | No | Air (separation, digestion) | - | - | ^31^ |
| SERS | AgNPs | PS (100, 500 nm), PE, PP (10 µm) | No | NaCl (-) | - | - | ^47^ |
| SERS | Ag-coated Au nanostars into porous Al_2_O_3_ | PS (0.4~4.8 µm) | No | Sea, tap, and river water (-) | - | - | ^48^ |
| SERS | AuNPs coated paper | PE (1~4 µm), PS (1µm) | No | River water, river (-) | logistic regression | - | ^42^ |
| Fluorescence | Peptide coupled to microenvironment-sensitive fluorophore unit | PNIPAM, PVP, PEG, and PVA | Yes | NaCl (-) | linear discriminant analysis | 94 | ^49^ |
| FTIR | - | PE, PP, PS, polyamide, cellulose acetate, Ethylene-vinyl acetate (>315 μm) | Yes | Sea waters (manual separation under a dissecting microscope) | Linear Discriminant Analysis, Support Vector Machines, Multilayer Perceptron, Random Forests classifier, K-Nearest Neighbors classification | ≤94 | ^24,28^ |
| Raman | - | PET, PP, PS, PVC, PE, and PC (10 µm) | No | - | Sparse autoencoder and soft-max classifier | 99.1 | ^50^ |
| SERS | 3D Ag foam coated by mesoAg and hydrophobic layer | PS, PE, PMMA, Nylon, PTFE (300 nm~90 µm) | Yes | Environmental matrix and wastewater (-) | SpecATNet | 100% -single MP;  ≈90% multi MP;  MP in env sample- 86.5 % | This work |

**Suppl. Table 6.** Information about the collected spectra and their amount used in this study.

| **Type of samples** | **Total № of measured spectra** | **Number of samples** |
| --- | --- | --- |
| MPs on glass and SiO_2_ | | |
| PE (glass) | 100 | 1 |
| PS (glass) | 100 | 1 |
| PS frag (glass) | 225 | 1 |
| PMMA (glass) | 100 | 1 |
| Nylon (glass) | 100 | 1 |
| PTFE (glass) | 102 | 1 |
| PET (glass) | 164 | 2 |
| PTFE | 100 | 1 |
| PE | 212 | 2 |
| PS | 144 | 1 |
| PMMA | 552 | 2 |
| Nylon | 112 | 1 |
| PTFE | 100 | 1 |
| PET | 144 | 2 |
| PS frag_deg_ | 680 | 3 |
| PS frag_deg_ (glass) | 64 | 1 |
| PET_deg_ | 500 | 4 |
| PE_deg_ | 100 | 1 |
| PS_deg_ | 120 | 1 |
| Multi component MPs mixtures | | |
| Nylon/PMMA | 725 | 2 |
| PE/PMMA | 742 | 3 |
| PS/PTFE | 243 | 1 |
| PMMA/PTFE | 866 | 2 |
| PS_deg_/PE_deg_ | 324 | 1 |
| PS/PE | 326 | 2 |
| PMMA/PS | 576 | 2 |
| PMMA/PTFE | 866 | 2 |
| PE/Nylon | 200 | 1 |
| PS/Nylon | 511 | 2 |
| PE/PTFE | 130 | 2 |
| PTFE/Nylon | 100 | 1 |
| PET fiber_deg_/PE | 284 | 3 |
| PET fiber_deg_/PS | 260 | 3 |
| PET fiber_deg_/PMMA | 385 | 3 |
| PET fiber/PTFE | 315 | 3 |
| PET fiber_deg_/Nylon | 382 | 3 |
| Nylon/PS/PTFE | 1394 | 2 |
| Nylon/PS/PMMA | 669 | 1 |
| PMMA/PS/PE | 1428 | 3 |
| PMMA/Nylon/PE | 720 | 1 |
| PMMA/PS/PTFE | 714 | 1 |
| PS/Nylon/PET | 180 | 1 |
| PE/PTFE/Nylon/PS/PMMA | 1800 | 4 |
| MPs on stored samples | | |
| PMMA/PS (on old AgF@AgM@C10) | 137 | 2 |
| PE/PTFE (on old AgF@AgM@C10) | 525 | 3 |
| Concentration dependence | | |
| 50 000 PL | 224 | 2 |
| 10 000 PL | 160 | 2 |
| 5 000 PL | 200 | 2 |
| 1 000 PL | 160 | 2 |
| 500 PL | 177 | 2 |
| 100 PL | 167 | 2 |
| 50 PL | 240 | 2 |
| 25 PL | 296 | 2 |
| 10 PL | 388 | 3 |
| Environmental samples | | |
| PE_deg_/PS_deg_ (simulated) | 574 | 1 |
| PE_deg_/PTFE_deg_ (simulated) | 604 | 1 |
| PS_deg_/PMMA_deg_ (simulated) | 980 | 2 |
| PMMA_deg_/Nylon_deg_ (simulated) | 382 | 2 |
| Nylon_deg_/PTFE_deg_ (simulated) | 288 | 1 |
| PMMA_deg_/PE_deg_ (wastewater) | 360 | 2 |
| PMMA_deg_/PE frag _deg_ (humic acid) | 470 | 2 |
| PTFE _deg_ /PE _deg_ (seawater+algae) | 882 | 3 |
| PET _deg_ /PE _deg_ frag (sediment) | 345 | 3 |
| PMMA _deg_ /PET _deg_ (humic acid+soil) | 297 | 3 |
| PS /PET _deg_ (algae) | 516 | 3 |
| PMMA/PE _deg_ /PS frag _deg_ (algae) | 276 | 1 |
| Nylon_deg_ /PS frag _deg_ (algae+sediment) | 204 | 2 |
| Negative control | | |
| AgF@AgM@C10 | 1039 | 6 |
| Groundwater | 3 | 456 |
| BSA/NaCl | 3 | 428 |
| Algae | 3 | 275 |
| Seawater | 2 | 213 |
| Sediment | 3 | 370 |
| Humic acid | 3 | 296 |
| Soil | 2 | 264 |

**Suppl. Table 7.** Composition of groundwater-certified reference material ERM-CA616.

| **Element** | **Mass Concentration** | |
| --- | --- | --- |
|  | Certified value ^a^ [mg L^-1^] | Uncertainty ^b^ [mg L^-1^] |
| Calcium | 42.6 | 1.4 |
| Chloride | 44.6 | 0.9 |
| Magnesium | 10.1 | 0.3 |
| Ortho-phosphate | 2.24 | 0.10 |
| Potassium | 5.79 | 0.15 |
| Sodium | 27.9 | 0.8 |
| Ammonium ^c^ | 0.583 | - |

^a^ Unweighted mean value of the means of accepted sets of data, each set being obtained in a different laboratory and/or with a different method of determination. The certified values and their uncertainties are traceable to the International System of Units (SI).

^b^ The certified uncertainty is the expanded uncertainty estimated in accordance with the Guide to the Expression of Uncertainty in Measurement (GUM, ISO/IEC Guide 98-3:2008) with a coverage factor k = 2, corresponding to a level of confidence of about 95 %

^c^ This is obtained by ion chromatography.

**Suppl. Table 8.** Calculation of cost of porous plasmonic substrate for 1 cm^2^ of foam (starting material only, based on prices on 30.10.2023).

| **Substrate** | **Price per cm^2^ (USD)** |
| --- | --- |
| Ag foam | 3.8 |
| Ni foam | 0.05 |
| Conductive soft PU foam | <0.01 |
| Mesoporous Ag | 0.09 |
| Mesoporous Au | 0.8* |
| Mesoporous Cu | 0.01* |
| Organic coating | 0.33 |

*Taking into account that precursor solution can be used two or three times

**Supplementary References**

1. Lim, H. *et al.* Synthesis of uniformly szed mesoporous silver films and their SERS application. *J. Phys. Chem. C* **124**, 23730–23737 (2020).

2. Prieto, P. *et al.* XPS study of silver, nickel and bimetallic silver–nickel nanoparticles prepared by seed-mediated growth. *Appl. Surf. Sci.* **258**, 8807–8813 (2012).

3. Moulder, J. F., Stickle, W. F., Sobol, P. E. ’, Bomben, K. D. & Chastain, J. Handbook of x-ray photoelectron spectroscopy: A reference book of standard spectra for identification and interpretation of XPS Data.

4. Gehan, H. *et al.* Thermo-induced electromagnetic coupling in gold/polymer hybrid plasmonic structures probed by surface-enhanced Raman scattering. *ACS Nano* **4**, 6491–6500 (2010).

5. Castle, J. E. Practical surface analysis by Auger and X-ray photoelectron spectroscopy. D. Briggs and M. P. Seah (Editors). John Wiley and Sons Ltd, Chichester, 1983, 533 pp., £44.50. *Surf. Interface Anal.* **6**, 302–302 (1984).

6. Ahmad, R. *et al.* Tailoring the surface chemistry of gold nanorods through Au-C/Ag-C covalent bonds using aryl diazonium salts. *J. Phys. Chem. C* **118**, 19098–19105 (2014).

7. Owens, D. K. & Wendt, R. C. Estimation of the surface free energy of polymers. *J. Appl. Polym. Sci.* **13**, 1741–1747 (1969).

8. Guselnikova, O. *et al.* SERS platform for detection of lipids and disease markers prepared using modification of plasmonic-active gold gratings by lipophilic moieties. *Sensors Actuators, B Chem.* **265**, 182–192 (2018).

9. Rius-Ayra, O., Biserova-Tahchieva, A. & LLorca-Isern, N. Surface-functionalised materials for microplastic removal. *Mar. Pollut. Bull.* **167**, 112335 (2021).

10. Han, Y. *et al.* Effect of oxidation on surface-enhanced raman scattering activity of silver nanoparticles: A quantitative correlation. *Anal. Chem.* **83**, 5873–5880 (2011).

11. Shim, W. J., Hong, S. H. & Eo, S. Marine microplastics: abundance, distribution, and composition. *Microplastic Contam. Aquat. Environ. An Emerg. Matter Environ. Urgency* 1–26 (2018).

12. Qi, H., Fu, D., Wang, Z., Gao, M. & Peng, L. Microplastics occurrence and spatial distribution in seawater and sediment of Haikou Bay in the northern South China Sea. *Estuar. Coast. Shelf Sci.* **239**, 106757 (2020).

13. Sagawa, N., Kawaai, K. & Hinata, H. Abundance and size of microplastics in a coastal sea: Comparison among bottom sediment, beach sediment, and surface water. *Mar. Pollut. Bull.* **133**, 532–542 (2018).

14. Chakraborty, I. *et al.* Raman spectroscopy for microplastic detection in water sources: a systematic review. *Int. J. Environ. Sci. Technol.* **20**, 10435–10448 (2023).

15. Bujacz, A. Structures of bovine, equine and leporine serum albumin. *Acta Cryst.* **D68**, 1278–1289 (2012).

16. Bujacz, A., Bujacz, G., Crystal structure of bovine serum albumin, PDB ID: 4F5S; https://doi.org/10.2210/pdb4F5S/pdb (2012)

17. Liu, P. *et al.* Effect of aging on adsorption behavior of polystyrene microplastics for pharmaceuticals: Adsorption mechanism and role of aging intermediates. *J. Hazard. Mater.* **384**, 121193 (2020).

18. Liu, Z. *et al.* Quantifying the dynamics of polystyrene microplastics UV-aging process. *Environ. Sci. Technol. Lett.* **9**, 50–56 (2022).

19. Silmeco nanopillar SERS substrate, substrate metal silver or gold. https://www.auroraprosci.com/Silmeco-Nanopillar-SERS-Substrate-Substrate-Metal-Silver-Gold.

20. Lim, H. *et al.* A universal approach for the synthesis of mesoporous gold, palladium and platinum films for applications in electrocatalysis. *Nat. Protoc.* **15**, 2980–3008 (2020).

21. La Nasa, J., Biale, G., Fabbri, D. & Modugno, F. A review on challenges and developments of analytical pyrolysis and other thermoanalytical techniques for the quali-quantitative determination of microplastics. *J. Anal. Appl. Pyrolysis* **149**, 104841 (2020).

22. David, J., Steinmetz, Z., Kučerík, J. & Schaumann, G. E. Quantitative analysis of poly(ethylene terephthalate) microplastics in soil via thermogravimetry-mass spectrometry. *Anal. Chem.* **90**, 8793–8799 (2018).

23. Back, H. de M., Vargas Junior, E. C., Alarcon, O. E. & Pottmaier, D. Training and evaluating machine learning algorithms for ocean microplastics classification through vibrational spectroscopy. *Chemosphere* **287**, 131903 (2022).

24. Kedzierski, M. *et al.* A machine learning algorithm for high throughput identification of FTIR spectra: application on microplastics collected in the Mediterranean Sea. *Chemosphere* **234**, 242–251 (2019).

25. Paul, A., Wander, L., Becker, R., Goedecke, C. & Braun, U. High-throughput NIR spectroscopic (NIRS) detection of microplastics in soil. *Environ. Sci. Pollut. Res.* **26**, 7364–7374 (2019).

26. Han, X.-L. *et al.* Deep learning based approach for automated characterization of large marine microplastic particles. *Mar. Environ. Res.* **183**, 105829 (2023).

27. Huang, H. *et al.* Proceeding the categorization of microplastics through deep learning-based image segmentation. *Sci. Total Environ.* **896**, 165308 (2023).

28. Yan, X., Cao, Z., Murphy, A. & Qiao, Y. An ensemble machine learning method for microplastics identification with FTIR spectrum. *J. Environ. Chem. Eng.* **10**, 108130 (2022).

29. S Maxwell, H., Melinda K, F. & Matthew, G. Counterstaining to separate Nile Red-stained microplastic particles from terrestrial invertebrate biomass. *Environ. Sci. Technol.* **54**, 5580–5588 (2020).

30. Velimirovic, M., Tirez, K., Voorspoels, S. & Vanhaecke, F. Recent developments in mass spectrometry for the characterization of micro- and nanoscale plastic debris in the environment. *Anal. Bioanal. Chem.* **413**, 7–15 (2021).

31. Xu, G. *et al.* Surface-enhanced Raman Spectroscopy facilitates the detection of microplastics <1 μm in the environment. *Environ. Sci. Technol.* **54**, 15594–15603 (2020).

32. Nuelle, M. T., Dekiff, J. H., Remy, D. & Fries, E. A new analytical approach for monitoring microplastics in marine sediments. *Environ. Pollut.* **184**, 161–169 (2014).

33. Fischer, M. & Scholz-Böttcher, B. M. Simultaneous trace identification and quantification of common types of microplastics in environmental samples by pyrolysis-gas chromatography-mass spectrometry. *Environ. Sci. Technol.* **51**, 5052–5060 (2017).

34. Primpke, S., Fischer, M., Lorenz, C., Gerdts, G. & Scholz-Böttcher, B. M. Comparison of pyrolysis gas chromatography/mass spectrometry and hyperspectral FTIR imaging spectroscopy for the analysis of microplastics. *Anal. Bioanal. Chem.* **412**, 8283–8298 (2020).

35. Luo, Y., Gibson, C. T., Tang, Y., Naidu, R. & Fang, C. Characterising microplastics in shower wastewater with Raman imaging. *Sci. Total Environ.* **811**, 152409 (2022).

36. Leonard, J. *et al.* Smartphone-enabled rapid quantification of microplastics. *J. Hazard. Mater. Lett.* **3**, 100052 (2022).

37. Dehaut, A. *et al.* Microplastics in seafood: Benchmark protocol for their extraction and characterization. *Environ. Pollut.* **215**, 223–233 (2016).

38. Ceccarini, A. *et al.* The hidden microplastics: new insights and figures from the thorough separation and characterization of microplastics and of their degradation byproducts in coastal sediments. *Environ. Sci. Technol.* **52**, 5634–5643 (2018).

39. Zhao, S., Danley, M., Ward, J. E., Li, D. & Mincer, T. J. An approach for extraction, characterization and quantitation of microplastic in natural marine snow using Raman microscopy. *Anal. Methods* **9**, 1470–1478 (2017).

40. Picó, Y. & Barceló, D. Pyrolysis gas chromatography-mass spectrometry in environmental analysis: Focus on organic matter and microplastics. *TrAC Trends Anal. Chem.* **130**, 115964 (2020).

41. Zhang, H. *et al.* 3D flexible SERS substrates integrated with a portable Raman analyzer and wireless communication for point-of-care application. *ACS Appl. Mater. Interfaces* **14**, 51253–51264 (2022).

42. Kim, J. Y. *et al.* 3D plasmonic gold nanopocket structure for SERS machine learning-based microplastic detection. *Adv. Funct. Mater.* **34**, 2307584 (2023).

43. Pyrolysis-GC-MS Analysis | Laboratory Services | Measurlabs. https://measurlabs.com/methods/pyrolysis-gc-ms-analysis/.

44. Identification of Microplastics with Raman Spectroscopy | Measurlabs. https://measurlabs.com/products/microplastic-identification-quantification-water-soil-sludge-raman-microscopy/.

45. Nie, X. L. *et al.* Recognition of plastic nanoparticles using a single gold nanopore fabricated at the tip of a glass nanopipette. *Chem. Commun.* **55**, 6397–6400 (2019).

46. Socrates, G. Infrared and Raman characteristic group frequencies : tables and charts. in 157, 191 (2001).

47. Lv, L. *et al.* In situ surface-enhanced Raman spectroscopy for detecting microplastics and nanoplastics in aquatic environments. *Sci. Total Environ.* **728**, 138449 (2020).

48. Lê, Q. T. *et al.* Nanostructured Raman substrates for the sensitive detection of submicrometer-sized plastic pollutants in water. *J. Hazard. Mater.* **402**, 123499 (2021).

49. Suzuki, S., Sawada, T. & Serizawa, T. Identification of water-soluble polymers through discrimination of multiple optical signals from a single peptide sensor. *ACS Appl. Mater. Interfaces* **13**, 55978–55987 (2021).

50. Luo, Y. *et al.* Raman spectroscopy and machine learning for microplastics identification and classification in water environments. *IEEE J. Sel. Top. Quantum Electron.* **29**, 6900308 (2023).
