# Supplementary material for: Pretreatment-free SERS sensing of microplastics using a self-attention-based neural network on hierarchically porous Ag foams
Source: Nat Commun. 2024 May 28;15:4351. doi: 10.1038/s41467-024-48148-w (PMC11133413; doi:10.1038/s41467-024-48148-w)
Supplement: Supplementary file 3 — Reporting Summary [file 41467_2024_48148_MOESM3_ESM.pdf]

Corresponding author(s): Yusuke Yamauchi, Joel Henzie, Olga Guselnikova

Last updated by author(s): Dec 14, 2023

Reporting Summary

Nature Portfolio wishes to improve the reproducibility of the work that we publish. This form provides structure for consistency and transparency in reporting. For further information on Nature Portfolio policies, see our Editorial Policies and the Editorial Policy Checklist.

Statistics

For all statistical analyses, confirm that the following items are present in the figure legend, table legend, main text, or Methods section.

n/a	Confirmed	
☒	☐	The exact sample size (*n*) for each experimental group/condition, given as a discrete number and unit of measurement
☐	☒	A statement on whether measurements were taken from distinct samples or whether the same sample was measured repeatedly
☒	☐	The statistical test(s) used AND whether they are one- or two-sided<br>*Only common tests should be described solely by name; describe more complex techniques in the Methods section.*
☒	☐	A description of all covariates tested
☒	☐	A description of any assumptions or corrections, such as tests of normality and adjustment for multiple comparisons
☐	☒	A full description of the statistical parameters including central tendency (e.g. means) or other basic estimates (e.g. regression coefficient) AND variation (e.g. standard deviation) or associated estimates of uncertainty (e.g. confidence intervals)
☒	☐	For null hypothesis testing, the test statistic (e.g. *F*, *t*, *r*) with confidence intervals, effect sizes, degrees of freedom and *P* value noted<br>*Give P values as exact values whenever suitable.*
☒	☐	For Bayesian analysis, information on the choice of priors and Markov chain Monte Carlo settings
☒	☐	For hierarchical and complex designs, identification of the appropriate level for tests and full reporting of outcomes
☒	☐	Estimates of effect sizes (e.g. Cohen's *d*, Pearson's *r*), indicating how they were calculated

Our web collection on statistics for biologists contains articles on many of the points above.

Software and code

Policy information about availability of computer code

Data collection	Spectra were collected on a JASCO 618 NRS3100 Raman spectrometer
Data analysis	https://github.com/Trel725/SpecATNet

For manuscripts utilizing custom algorithms or software that are central to the research but not yet described in published literature, software must be made available to editors and reviewers. We strongly encourage code deposition in a community repository (e.g. GitHub). See the Nature Portfolio guidelines for submitting code & software for further information.

Data

Policy information about availability of data

All manuscripts must include a data availability statement. This statement should provide the following information, where applicable:

- Accession codes, unique identifiers, or web links for publicly available datasets
- A description of any restrictions on data availability
- For clinical datasets or third party data, please ensure that the statement adheres to our policy

All data and code needed to replicate these results are available at https://www.kaggle.com/datasets/andriitrelin/microplastics-raman-spectra

Research involving human participants, their data, or biological material

Policy information about studies with human participants or human data. See also policy information about sex, gender (identity/presentation), and sexual orientation and race, ethnicity and racism.

Reporting on sex and gender	N/A
Reporting on race, ethnicity, or other socially relevant groupings	N/A
Population characteristics	N/A
Recruitment	N/A
Ethics oversight	N/A

Note that full information on the approval of the study protocol must also be provided in the manuscript.

Field-specific reporting

Please select the one below that is the best fit for your research. If you are not sure, read the appropriate sections before making your selection.

☐ Life sciences ☐ Behavioural & social sciences ☒ Ecological, evolutionary & environmental sciences

For a reference copy of the document with all sections, see nature.com/documents/nr-reporting-summary-flat.pdf

Ecological, evolutionary & environmental sciences study design

All studies must disclose on these points even when the disclosure is negative.

Study description	Microplastic in water sample was analyzed by surface-enhanced Raman spectroscopy and neural networks
Research sample	Microplastics (polystyrene, polyethylene, polymethylmethacrylate, polytetrafluoroethylene, nylon, polyethylene terephthalate) in water and aqueous suspension of soil, algae, humic acid, protein, marine sediments, synthetic seawater
Sampling strategy	Environmental samples of microplastics were not collected or sampled, commercially available samples of analytical standart were used.
Data collection	Spectra were collected on a JASCO 618 NRS3100 Raman spectrometer
Timing and spatial scale	Spectra were collected from January 2022 to November 2023. During every experiment, freshly prepared suspensions of microplastics were used.
Data exclusions	No data was excluded.
Reproducibility	Full dataset and codes are available at https://www.kaggle.com/datasets/andriitrelin/microplastics-raman-spectra and https://github.com/Trel725/SpecATNet to reproduce our findings. For other data, such as Raman spectra, wettability, surface free energy and adsorption capacity measurement, the standard deviation was calculated
Randomization	Six microplastics were mixed into different compositions (e.g. 2 types, 3 types, 4 types or 5 types of microplastic) or type of sample by simple randomization method taking into account the need to reach a balanced dataset. Balanced datasets were achieved by ensuring each microplastic has a similar number of Raman spectra collected on each sample.
Blinding	No direct blinding was used because the data was split into training and test sets in a 10-fold cross-validation manner to train our neural network. In simple terms, the neural network had no inkling of what sample it was measuring.

Did the study involve field work? ☐ Yes ☒ No

Reporting for specific materials, systems and methods

We require information from authors about some types of materials, experimental systems and methods used in many studies. Here, indicate whether each material, system or method listed is relevant to your study. If you are not sure if a list item applies to your research, read the appropriate section before selecting a response.

Materials & experimental systems

n/a	Involved in the study
☒	☐ Antibodies
☒	☐ Eukaryotic cell lines
☒	☐ Palaeontology and archaeology
☒	☐ Animals and other organisms
☒	☐ Clinical data
☒	☐ Dual use research of concern
☒	☐ Plants

Methods

n/a	Involved in the study
☒	☐ ChIP-seq
☒	☐ Flow cytometry
☒	☐ MRI-based neuroimaging

Plants

Seed stocks	n/a
Novel plant genotypes	n/a
Authentication	n/a
